# Next-Generation Hydrogels Integrating Natural Antioxidants and Microbiome Modulators for Improved Cancer Management

**DOI:** 10.3390/gels12030249

**Published:** 2026-03-16

**Authors:** Camelia Munteanu, Eftimia Prifti, Larisa Achim, Ciprian Nicolae Silaghi, Sorin Marian Mârza

**Affiliations:** 1 Biology Section, Faculty of Agriculture, University of Agricultural Sciences and Veterinary Medicine Cluj-Napoca, 3-5 Manastur Street, 400372 Cluj-Napoca, Romania; eftimia.prifti@student.usamvcluj.ro (E.P.); larisa-daniela.achim@student.usamvcluj.ro (L.A.); 2 Molecular Sciences Department, University of Medicine and Pharmacy “Iuliu Hatieganu”, 400349 Cluj-Napoca, Romania; silaghi.ciprian@umfcluj.ro; 3 Clinical Sciences Department, Faculty of Veterinary Medicine, University of Agricultural Sciences and Veterinary Medicine Cluj-Napoca, 3-5 Manastur Street, 400372 Cluj-Napoca, Romania; sorin.marza@usamvcluj.ro

**Keywords:** antioxidants, prebiotics, probiotics, synbiotics, cancer, hydrogels

## Abstract

Cancer remains a leading cause of death worldwide, and current treatments are often limited by toxicity and resistance. Emerging research highlights the crucial roles played by gut microbiome dysbiosis and oxidative stress in cancer development and treatment response. Through their antioxidant, anti-inflammatory, and immunomodulatory properties, natural antioxidants such as resveratrol, along with microbiome modulators like probiotics, prebiotics, and synbiotics, offer promising therapeutic benefits. However, issues such as low bioavailability, instability, and challenges related to targeted delivery hinder the clinical translation of these bioactive compounds. Next-generation hydrogels have emerged as adaptable platforms capable of delivering and protecting these agents in a site-specific and controlled manner. This review summarizes the design and synthesis of multifunctional hydrogels incorporating natural antioxidants and microbiome modulators for cancer therapy.

## 1. Introduction

Cancer is still one of the biggest medical problems facing the globe today and a major cause of death. The overall effectiveness and long-term success of traditional treatments, including chemotherapy, radiation, immunotherapy, and targeted molecular therapies, are often limited by substantial obstacles despite notable breakthroughs in these treatments. These include the quick and frequently unavoidable development of acquired or intrinsic drug resistance, high systemic toxicity that restricts the maximum acceptable dose, and an inadequate ability to discriminate between malignant and healthy tissues [1]. Furthermore, therapy failure or patient recurrence are frequently caused by the varied character of tumors and the complexity of the tumor microenvironment (TME). The necessity to investigate and create new therapy strategies that are intrinsically safer, more effective, show more specificity, and target several disease pathways at once is highlighted by these enduring clinical obstacles [2]. Nanotechnology and intelligent drug delivery systems, which can enhance therapeutic impact while reducing collateral damage, are frequently the result of this exploration [3].

Furthermore, oxidative stress is one of the major biological processes linked to the development, spread, and resistance to therapy of cancer. An imbalance between the body’s antioxidant defense systems and the generation of reactive oxygen species (ROS) results in oxidative stress. Excessive ROS can cause DNA damage, genomic instability, lipid peroxidation, and protein malfunction, all of which contribute to cancer, even though reasonable ROS levels are crucial for healthy cellular communication and immunological responses [4]. Because of their high metabolic activity and disturbed redox balance, cancer cells frequently display higher ROS levels. Uncontrolled oxidative stress also encourages tumor growth, metastasis, and resistance to chemotherapy and radiation, even though this vulnerability can be used therapeutically. As a result, methods for controlling oxidative stress—rather than just getting rid of ROS—are becoming more widely acknowledged as essential elements of successful cancer treatment [5].

In order to control redox equilibrium and shield healthy tissues from oxidative damage, natural antioxidants have garnered a lot of interest as potential therapeutic agents in the management of cancer [6]. Strong antioxidant, anti-inflammatory, and anticancer effects are shown by substances such polyphenols, flavonoids, carotenoids, vitamins, and bioactive compounds produced from plants. Natural antioxidants have been shown in numerous studies to decrease angiogenesis, induce apoptosis, inhibit the growth of cancer cells, and increase the sensitivity of tumor cells to traditional therapies [7]. They are also appealing options for combination therapy and long-term use due to their usually positive safety ratings. However, poor solubility, low bioavailability, fast breakdown, and non-specific distribution frequently limit the clinical use of natural antioxidants. These difficulties demonstrate the need for sophisticated delivery systems that can safeguard these substances and guarantee their targeted and regulated release at tumor sites [8].

Moreover, the gut microbiota has become a critical regulator of cancer formation, progression, and treatment outcomes in tandem with oxidative stress. Trillions of bacteria make up the human gut microbiota, which is essential for immunological homeostasis, metabolic balance, and epithelial integrity [9]. Immune dysregulation, chronic inflammation, and an increased risk of colorectal, liver, breast, and lung malignancies have all been associated with dysbiosis, an imbalance in the composition and function of the gut microbiome. Although certain microbial communities could promote resistance to medicines or adverse effects, certain ones can enhance anticancer immune responses [10,11].

Prebiotics, probiotics, postbiotics, and bioactive dietary ingredients are examples of microbiome modulators that present potential chances to improve cancer treatment outcomes and restore microbial equilibrium [12]. Microbiome modulators can improve the body’s natural defenses against cancer, strengthen immunological surveillance, and reduce inflammation by selectively fostering beneficial microbial communities and reducing harmful species [13]. Additionally, interactions between the gut microbiome and naturally occurring antioxidants point to a synergistic relationship since microbial metabolism can affect the availability and bioactivity of antioxidant molecules, while antioxidants can influence the composition and function of microbes [13].

Hydrogels have become extremely adjustable and adaptable biomaterials for tissue engineering and drug administration in this changing therapeutic landscape [3]. Hydrogels are three-dimensional networks of hydrophilic polymers that can hold a lot of water without losing their structural integrity. They are especially well suited for biomedical applications due to their biocompatibility, biodegradability, and controlled release capability [3]. Therapeutic compounds can be delivered site-specifically and on demand thanks to the ability of next-generation hydrogels to react to particular stimuli like pH, temperature, enzymes, or redox conditions [3].

An innovative and promising strategy for better cancer treatment is the incorporation of microbiome modulators and natural antioxidants into sophisticated hydrogel systems. These multipurpose hydrogels can distribute bioactive substances locally and sustainably, shield them from deterioration, and improve their therapeutic efficacy while lowering systemic negative effects. Furthermore, these novel platforms address important interrelated pathways driving cancer progression by concurrently addressing oxidative stress and microbiome dysbiosis [14].

In order to develop next-generation hydrogel platforms that include natural antioxidants and microbiome modulators, this study aims to present a thorough and mechanistically grounded examination of context-dependent redox regulation in cancer therapy. In particular, this study aims to elucidate the dual function of reactive oxygen species (ROS) in cancer biology, emphasizing how excessive ROS can be used therapeutically to kill cancer cells while chronic and dysregulated oxidative stress encourages tumor growth and resistance to treatment. This puts antioxidant-based approaches in a position where they are complementary and context-specific rather than antagonistic. Precision redox regulation and microbiome modulation inside sophisticated hydrogel-based delivery systems are the main topics of this review, which tackles the urgent need for safer, more efficient, and mechanistically informed cancer therapeutics. The review’s objective is to investigate the ways in which natural antioxidants and microbiome modulators, especially when administered through multifunctional, stimuli-responsive hydrogel systems, can modify inflammation, restore redox balance, and affect immunological responses. The novelty of this paper is due to the fact that it examines three interrelated therapeutic dimensions: microbiome modulators as important regulators of inflammation, immune responses, and treatment efficacy; multifunctional, stimuli-responsive hydrogels as sophisticated platforms that allow for the targeted, controlled, and synergistic delivery of these bioactive agents; and natural antioxidants as modulators of redox balance rather than simple ROS scavengers.

In order to create a cohesive view of precision redox regulation as a tactic to improve therapeutic efficacy, lower systemic toxicity, and overcome resistance in cancer treatment, this review aims to integrate developments in biomaterials science with new knowledge about oxidative stress and gut microbiome interactions.

Methods:

This review evaluates advances in hydrogel-based delivery systems that incorporate natural antioxidants and microbiome modulators for cancer therapy. The methodology systematically identifies, analyzes, and synthesizes current scientific evidence regarding antioxidant-loaded and microbiome-responsive hydrogels, with particular emphasis on their design principles, biological mechanisms, and therapeutic relevance in oncology.

*Search for Literature and Selection Method:*

A comprehensive and systematic literature search was performed to identify relevant peer-reviewed publications, such as research articles, review papers, and selected clinical studies. The search included electronic databases such as PubMed, Web of Science, Scopus, ScienceDirect, and Google Scholar to ensure extensive coverage of biomedical, pharmaceutical, and materials science literature.

The primary keywords included: “hydrogels”, “smart hydrogels”, “stimuli-responsive hydrogels”, “injectable hydrogels”, “natural antioxidants”, “polyphenols”, “catechins”, “EGCG”, “lycopene”, “β-glucans”, “resveratrol”, “probiotics”, “prebiotics”, “synbiotics”, “gut microbiome”, “microbiota modulation”, “cancer therapy”, “tumor microenvironment”, “oxidative stress”, “controlled drug delivery”, “site-specific delivery”, “pH-responsive release”.

*Classification of Hydrogel Systems*

Hydrogel systems discussed in this review were categorized based on polymer origin: natural (e.g., chitosan, alginate, gelatin), synthetic (e.g., PEG, PVA), and hybrid systems; responsiveness to stimuli: pH, temperature, redox conditions, enzymatic activity, or microbial metabolites, and therapeutic function: antioxidant delivery, microbiome modulation, immune activation, or combination therapy.

*Sources of natural antioxidants:*

The review analyzes data extracted from peer-reviewed studies relevant to antioxidant- and microbiome-modulating hydrogel systems for cancer therapy. The analyzed data include the types and sources of natural antioxidants and microbiome modulators, mechanisms of anticancer activity (such as ROS regulation, apoptosis induction, immune modulation, and microbiome-mediated effects).

*Hydrogel Performance and Outcomes*

The review also assesses drug delivery performance parameters (loading efficiency, release kinetics, stability, and targeting), hydrogel fabrication and crosslinking techniques, and therapeutic outcomes (anticancer efficacy, immunomodulation, microbiome regulation, and toxicity profiles).

*Methods for Figures*

*Figures were created in BioRender. Munteanu, C. (2026) https://BioRender.com/g5lgjmw, https://BioRender.com/wft23gi/ and https://BioRender.com/1a0z8d1 (accessed on 2 March 2026).*

## 2. Natural Antioxidants in Cancer Therapy

Several natural antioxidants have demonstrated significant strategy in cancer prevention and therapy through their ability to regulate reactive oxygen species (ROS), which are critically involved in tumor development and treatment response [15,16]. Dietary-derived bioactive compounds such as polyphenols, carotenoids, and vitamins can protect normal cells from oxidative stress while promoting apoptosis in malignant cells [17].

These compounds, commonly classified as nutraceuticals, exert pleiotropic effects by modulating key cellular signaling pathways and display a favorable safety profile compared with conventional cytotoxic agents. In light of the increasing global cancer burden and the limitations of standard therapies, including systemic toxicity and acquired drug resistance, natural antioxidant-based approaches are being increasingly explored as complementary options in oncology [17,18].

### 2.1. Key Natural Antioxidants with Anticancer Properties

Probiotics and prebiotics are examples of natural antioxidants and microbiome modulators that offer a promising approach to addressing processes involved in carcinogenesis. Epigallocatechin-3-gallate (EGCG), lycopene, and β-glucans are examples of compounds that control oxidative stress, cause cancer cells to undergo apoptosis, and alter the composition of the gut microbiota. However, their clinical application is limited by poor solubility, low gastrointestinal stability, rapid systemic clearance, and limited bioavailability [19,20,21]. A possible solution is provided by hydrogel-based delivery systems, which allow these bioactives to be co-encapsulated, shielded from deterioration, and released under regulated, site-specific conditions. This method enhances therapeutic concentrations in the tumor microenvironment and facilitates multifunctional approaches that target the composition of the gut bacteria as well as tumor redox balance [22,23,24].

Although natural antioxidants and microbiota modulators target interconnected pathways implicated in cancer growth, their combined use offers more therapeutic potential than single-agent methods. While microbiome modulators affect immune surveillance and microbial metabolites that impact tumor biology, antioxidants control oxidative stress and inflammation [25]. Antioxidants alter microbial composition, and gut microbiota can alter antioxidant metabolism and bioavailability. A synergistic impact that increases anticancer activity is produced by this reciprocal interaction [13].

EGCG is the most abundant and biologically potent polyphenolic constituent identified in green tea (*Camellia sinensis*) [26]. It has potent anticancer potential through antioxidant effects and regulation of signaling pathways such as MAPK, PI3K/Akt/mTOR, and NF-κB, which causes apoptosis and inhibits tumor growth [20]. The presence of this specific gallic acid group is the primary determinant of its superior antioxidant and antiproliferative efficacy relative to other catechins like epicatechin (EC) or epigallocatechin (EGC) [20,26,27,28]. In vitro studies across diverse cancer cell lines, including pancreatic (Panc-1, MIA PaCa-2), colon (HCT15, SW480), and lung (A549, H358), have demonstrated that EGCG reduces cancer cell growth in a time- and concentration-dependent manner. Furthermore, EGCG acts as a modulator of the TME by inhibiting vascular endothelial growth factor (VEGF) expression and acting as a potential programmed death-ligand 1 (PD-L1) inhibitor [29,30,31,32].

The lipophilic carotenoid lycopene (*Solanum lycopersicum*), which is mostly present in tomatoes, has strong antioxidant properties and promotes pro-apoptotic signaling while reducing oxidative stress, inflammation, and the growth of cancer cells. Lycopene’s anticancer mechanisms involve the regulation of redox homeostasis, suppression of chronic inflammation, and induction of pro-apoptotic signaling pathways [21,33,34]. Despite its high therapeutic potential, lycopene’s extreme hydrophobicity and susceptibility to isomerization and oxidation necessitate its delivery via advanced encapsulation systems to improve bioaccessibility [33,34,35].

β-Glucans are complex polysaccharides composed of D-glucose monomers linked by β-(1 to 3), (1 to 4), or (1 to 6) glycosidic bonds [36]. They are structural components of fungal cell walls (*Poria cocos*, *Saccharomyces cerevisiae*), yeast, cereals (oats, barley), and certain bacteria. The bioactivity of β-glucans is intrinsically linked to their structural features, including molecular weight, branching frequency, and tertiary conformation (e.g., triple-helix) [37,38,39,40]. Their capacity to suppress tumor growth and alter the tumor microenvironment is further supported by preclinical research [41,42]. Additionally, β-glucans provide antioxidant protection by scavenging excess ROS and boosting endogenous enzymes, reducing mutagenesis risk and safeguarding healthy tissues from therapy-induced damage [43] (Table 1).

### 2.2. Mechanisms of Action and Therapeutic Potential (Catechins and Flavonoids, Epigallocatechin-3-Gallate (EGCG), Carotenoid Lycopene and Antioxidant Polysaccharide β-Glucans)

**Figure 1:**
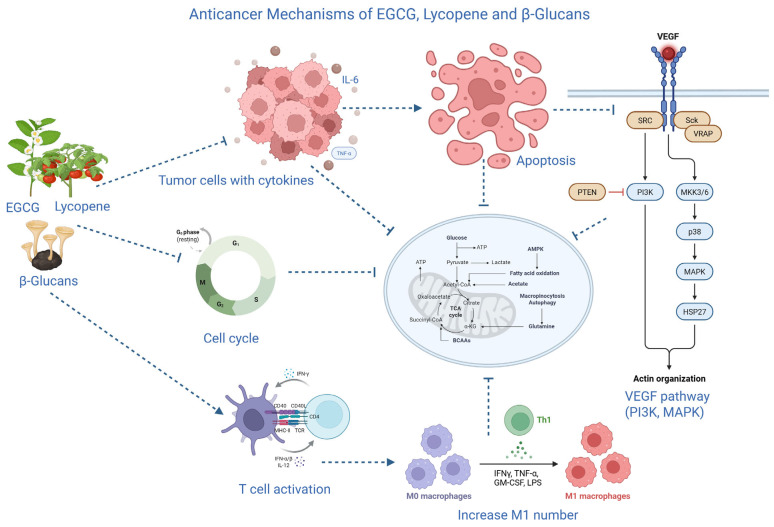
Anticancer mechanisms of EGCG, lycopene, and β-glucans. Epigallocatechin gallate (EGCG) and lycopene exert anticancer effects primarily through modulation of tumor cell signaling, oxidative stress, and inflammatory responses. These compounds inhibit pro-tumorigenic cytokine production (e.g., IL-6, TNF-α), suppress cell cycle progression, interfere with cancer cell metabolism, and promote apoptosis. In addition, EGCG and lycopene inhibit angiogenesis-related signaling pathways, including VEGF-mediated PI3K/Akt and MAPK pathways. β-Glucans act mainly as biological response modifiers by activating innate and adaptive immune responses. They enhance antigen presentation and T-cell activation, promote macrophage polarization toward the antitumoral M1 phenotype, and contribute to immune-mediated tumor cell elimination. Collectively, these mechanisms converge to inhibit tumor growth, induce apoptosis, and remodel the tumor microenvironment toward an antitumor state.

1. The anticancer effects of EGCG are mediated through multiple signaling pathways, with its antioxidant properties playing a supportive role by reducing oxidative stress that drives cancer progression [44,45]. A key mechanism involves modulation of the Mitogen-Activated Protein Kinase (MAPK) and Phosphoinositide 3-kinase (PI3K)/Akt pathways. In pancreatic cancer cells, EGCG inhibits phosphorylation of Extracellular signal-regulated kinases 1 and 2 (ERK1/2) while activating pro-apoptotic JNK and p38 MAPK, leading to cell cycle arrest and apoptosis [45,46,47]. In addition, EGCG induces G1 phase arrest by up-regulating CDK inhibitors Cyclin-dependent kinase inhibitor 1A (p21Cip1/Waf1) and Cyclin-dependent kinase inhibitor 1B (p27KIP1), and down-regulating cyclin D1, Cdk4, and Cdk6 [26,48]. Furthermore, EGCG exerts anti-inflammatory effects by inhibiting NF-κB: blocking IκBα phosphorylation/degradation, reducing pro-inflammatory cytokines (IL-6, TNF-α) [46,49,50].

2. The therapeutic potential of lycopene in cancer is primarily driven by its exceptional antioxidant activity the strongest singlet oxygen quencher among carotenoids, and downstream effects that mitigate oxidative stress, a key driver of cancer initiation, progression, and inflammation [51,52,53]. A distinctive mechanism is lycopene’s upregulation of gap junctional intercellular communication (GJC) via increased transcription and expression of Connexin 43 (Cx43), often downregulated in cancers (e.g., oral, breast, prostate) [51,54,55]. Lycopene modulates oncogenic pathways, including inhibition of Wnt/β-catenin (preventing nuclear translocation and EMT-related gene activation in colorectal models) and PI3K/Akt/mTOR [53].

3. Unlike direct antioxidants such as EGCG or lycopene, the anticancer therapeutic potential of β-glucans stems from their role as Biological Response Modifiers (BRMs), which orchestrate a robust host-mediated antitumor immune response by activating innate and adaptive immunity [56,57,58]. Immune activation is often stronger for β-glucans with higher molecular weight and more branches, while changes to the backbone or (1→6) side chains can reduce biological activity and receptor recognition. This link between structure and activity emphasizes how crucial accurate β-glucan characterisation is to maximizing therapeutic results [59]. Binding to (1→3)-β-D-glucans triggers a Syk-dependent cascade, leading to: Enhanced phagocytosis of tumor cells; Oxidative burst with production of ROS and nitric oxide (NO) for direct killing; Secretion of pro-inflammatory cytokines (TNF-α, IL-1β, IL-6, IL-12) to recruit immune cells [57,58,60,61,62]. [Additionally, β-glucans bridge innate and adaptive immunity by maturing DCs, improving antigen presentation to CD8^+^ cytotoxic and CD4^+^ helper T cells, and fostering tumor-specific T-cell responses. Clinical evidence shows that compounds like Imprime PGG enhance monoclonal antibody therapies (e.g., rituximab, pembrolizumab) by shifting the tumor microenvironment toward immune stimulation, with increased M1 macrophages and activated tumor-infiltrating lymphocytes [56] (Figure 1). Hydrogel encapsulation bridges the gap between preclinical efficacy and possible clinical application by ensuring regulated, localized release and shielding labile chemicals from degradation.

In terms of quantitatively characterizing hydrogel delivery systems, a number of studies have documented important performance characteristics for hydrogels loaded with natural antioxidants, including loading efficiency, release patterns, and degradation behavior. As an example of how encapsulation depends on hydrogel formulation factors, EGCG has been effectively added to polymeric hydrogels with loading efficiencies exceeding ~67% under ideal crosslinking circumstances [28]. Alginate and pectin-based oral hydrogel carriers demonstrated both quantitative loading and in vivo release behavior in other designs, achieving high encapsulation efficiencies for EGCG and other phenolic compounds (up to ~92%) with targeted release in the intestinal tract after oral administration [63]. For antioxidant-embedded hydrogel systems, controlled release kinetics have also been reported. For instance, lycopene loaded in a HAMA–GelMA hydrogel showed sustained diffusion over 96 h, releasing only about 24% in the first 6 h and 74% cumulatively by 96 h, indicating a significant modulation of release in comparison to free compounds [64]. Studies have measured the hydrogel matrix’s disintegration over time in physiological settings in order to understand degradation behavior. One EGCG-loaded network showed a slow decrease in mass in vitro, with full degradation taking place over around 20 days and continuous cargo release during that time [65]. In spite of the lack of systematic comparative data for cancer models and across hydrogel classes, these findings show that sophisticated hydrogel formulations can achieve defined encapsulation efficiencies, regulated release profiles spanning hours to weeks, and predictable degradation patterns.

### 2.3. Challenges in Clinical Application

The translation of natural antioxidants (e.g., EGCG, lycopene, and others) from promising preclinical (in vitro and animal) data to clinical oncology practice is severely limited by poor bioavailability, metabolic instability, and lack of product standardization, resulting in suboptimal systemic exposure and inconsistent therapeutic outcomes [20,66,67].

1. For EGCG, oral bioavailability is typically <10%, driven by:

Environmental instability: Rapid auto-oxidation and degradation in the alkaline small intestine (pH ~ 8) versus stability in acidic stomach (pH ~ 2), due to vulnerability of the pyrogallol ring leading to hydrogen peroxide formation and structural loss.

Poor membrane permeability: High hydrophilicity from eight hydroxyl groups hinders passive diffusion across lipid-rich intestinal membranes.

Rapid clearance and metabolism: Active efflux by P-glycoprotein (P-gp) back into the lumen; extensive phase II hepatic conjugation (methylation, sulfation, glucuronidation) producing less active metabolites [20,48,68].

These factors contribute to high interindividual variability in plasma levels, complicating dose standardization and reproducible efficacy. Emerging strategies include nanocarriers, liposomes, and prodrugs to protect against pH degradation, improve lipophilicity, and bypass efflux transporters [20,69].

For lycopene, the clinical challenges are centered on its absolute dependency on the food matrix and dietary fat for absorption, as well as its inherent lipophilicity which limits its distribution in aqueous physiological environments [70,71]. The clinical failure of these compounds to date is largely a failure of delivery, not mechanistic capacity. The therapeutic doses used in successful in vitro studies are rarely achieved in human plasma due to the physiological barriers described. Therefore, the future of these antioxidants in oncology lies in advanced pharmaceutical engineering. The development of targeted nanocarriers such as EGCG-functionalized iron oxide nanoparticles or hollow β-glucan spheres offers a way to bypass the digestive environment and deliver these potent compounds directly to the tumor microenvironment or to specific immune cell populations [67,72].

2. Lycopene’s absorption is “matrix-bound”. In fresh tomatoes, it is sequestered within plant chromoplasts and associated with dietary fiber, which can reduce its absorption by up to 40%. The transition from the all-*trans* isomer found in nature to the more bioavailable *cis*-isomers requires thermal processing. However, excessive heat or air-drying can lead to oxidative degradation, creating a narrow window for optimal processing. Furthermore, as a highly lipophilic compound, lycopene absorption requires the presence of dietary lipids to facilitate the formation of mixed micelles in the small intestine. Clinical studies have shown a dose–response relationship between fat intake and lycopene absorption, with negligible absorption occurring in the absence of dietary fat [73,74].

Another layer of complexity is the interaction with other dietary components and genetic factors. Lycopene absorption is inhibited by the presence of high-fiber diets and competing carotenoids like β-carotene. On a genetic level, single nucleotide polymorphisms (SNPs) in the scavenger receptor class B type 1 (SR-B1) gene and the intestine-specific homeobox (ISX) transcription factor can significantly alter an individual’s ability to absorb lycopene. These factors lead to a “fragmented” evidence base where observational studies and supplementation trials often yield inconsistent results regarding clinical outcomes like glycemic control or metabolic biomarkers [60,71,75,76].

3. Aggressive chemical extraction methods (e.g., hot alkali treatment) can degrade the β-(1,3) backbone or strip away the β-(1,6) side chains that are essential for Dectin-1 and CR3 receptor binding. Conversely, crude extracts often contain “impurities” such as proteins, mannans, and lipids, which can trigger unintended inflammatory responses or even septic shock if the dose is not carefully controlled. This has historically led to a preference for intravenous or intraperitoneal administration in research, although modern studies suggest that oral β-glucans are slowly degraded by splenic macrophages and released as active fragments into the systemic circulation [57,61,77].

Standardization of dosing and purity is currently lacking. No “gold standard” purified β-glucan has been universally adopted for clinical use, and there is a significant need for large-scale randomized clinical trials to determine the optimal molecular weight and branching frequency for different cancer types. Furthermore, potential interference in diagnostic assays such as false-positive results in fungal infection tests for patients receiving β-glucan therapy poses a practical challenge for oncologists managing complex patient cases [36,78].

Furthermore, the synergistic potential of these agents with conventional therapies, such as EGCG’s ability to reverse P-gp-mediated multidrug resistance and β-glucan’s capacity to enhance the effectiveness of checkpoint inhibitors, suggests that they should be viewed as essential components of a multimodal treatment strategy rather than stand-alone cures. As our understanding of structural-activity relationships and the role of the gut microbiota in metabolite activation continues to expand, these natural antioxidants are poised to transition from “dietary supplements” to validated, high-potency pharmacological tools in the fight against cancer. However, potential risks related to immune overstimulation by β-glucans should not be overlooked, especially in individuals with pre-existing immune dysregulation. Careful dose optimization and monitoring are warranted to maximize therapeutic benefits while minimizing adverse immune effects. The noble goal of this research is to create a more resilient host environment, where the natural and the synthetic work in tandem to achieve durable remission and improved quality of life for patients [57,79,80].

The application of hydrogel-based delivery systems tackles important therapeutic issues such as EGCG, lycopene, and β-glucans’ limited tissue targeting, fast metabolism, and poor bioavailability. Hydrogels improve the pharmacokinetic and pharmacodynamic profiles of these bioactives by permitting site-specific release and sustained local concentrations. They also provide co-delivery with microbiota modulators to produce synergistic anticancer effects (Table 2).

### 2.4. Comparison with Conventional Systemic Delivery Strategies

Sub-therapeutic tumor exposure and uneven clinical results are the results of conventional systemic administration of natural antioxidants, such as oral or intravenous delivery of EGCG, lycopene, or β-glucans, which is severely limited by poor bioavailability, rapid metabolism, and non-specific distribution. For instance, lycopene absorption is heavily reliant on dietary fat and food matrix effects, whereas oral EGCG has a plasma bioavailability of less than 10%, mostly because of instability in the small intestine, significant phase II metabolism, and P-glycoprotein-mediated efflux. Although intravenous or intraperitoneal β-glucan treatment avoids the gut, it has drawbacks, including quick clearance, possible off-target immune activation, and a lack of consistent dose [81].

Advanced hydrogel and nanoparticle-based delivery systems, on the other hand, successfully get around the drawbacks of traditional systemic approaches by enabling site-specific, controlled release, protection against oxidative or enzymatic degradation, improved tumor penetration, and increased local concentration. Compared to oral or intravenous methods, quantitative studies provide more dependable pharmacokinetics and pharmacodynamics because to loading efficiencies of up to 90%, prolonged release over several days, and predictable degradation kinetics [82]. These tailored delivery methods are therefore viable substitutes or supplements to conventional systemic medicines because they provide increased therapeutic efficacy, decreased systemic toxicity, and greater repeatability.

Hydrogel-based delivery of antioxidants and microbiota modulators offers finer geographic and temporal control of release, better protection against degradation, and improved interaction with the tumor microenvironment as compared to traditional systemic administration. By minimizing systemic toxicity and optimizing therapeutic efficacy, this approach demonstrates the translational potential of multifunctional hydrogel carriers.

## 3. Microbiome Modulators in Oncology

### 3.1. Probiotics, Prebiotics, and Synbiotics: Definitions and Roles

The gut microbiome influences cancer in two ways by impacting both the development of cancer and treatment results through mechanisms like inflammation, immune system changes, metabolic activities, and genotoxic effects [83]. The microbiome plays a vital, foundational role in maintaining overall health; however, microbial dysbiosis can shift a diverse bacterial community toward a maladaptive, pathogenic state [84,85]. This alteration has been associated with a range of diseases, such as diabetes, cardiovascular disease, and multiple forms of cancer [13,86,87].

Probiotics suppress harmful microorganisms and modify the intestinal microbial composition, thereby supporting a healthier host population [88]. Following colonization, probiotics induce immune responses by stimulating immunoregulatory molecules in intestinal epithelial cells. Furthermore, metabolites produced by probiotics, such as bacteriocins, amines, and hydrogen peroxide, modulate metabolic pathways that govern apoptosis, cell proliferation, inflammation, and cellular differentiation [11,89]. Importantly, numerous randomized clinical trials have demonstrated that probiotic strains are both safe and effective [90,91,92]. Interestingly, synbiotics were developed to address the survival challenges that probiotics encounter during transit through the upper intestine [92,93]. Synbiotics modulate gut metabolism, maintain gut structural integrity, and elevate levels of compounds such as short-chain fatty acids and ketones, which are associated with potential health-promoting effects [94,95,96]. Moreover, prebiotics such as *fructooligosaccharides*, *galactooligosaccharides*, and *xylooligosaccharides* promote the growth of beneficial bacteria already present in the colon and enhance the activity of probiotic bacteria introduced from outside the body [90,97,98] (Table 3). By selectively stimulating specific microbial groups, they help create positive changes in the composition and activity of the gut microflora. Through these targeted effects on the gastrointestinal ecosystem, prebiotics ultimately contribute to improved physiological status [91].

### 3.2. Impact on Cancer Development and Treatment Response

The microbiota is essential for the maturation of the immune system and the initiation of anticancer responses, both in the gut and at distant anatomical sites. Dendritic cells, natural killer (NK) cells, and T cells function as primary effectors in detecting and eliminating damaged or potentially carcinogenic host cells. Probiotics engage dendritic cells via cell-surface pattern recognition receptors, such as toll-like receptors, thereby stimulating T cell and NK cell responses. Synbiotic and probiotic formulations containing *Lactobacillus casei* or *Bifidobacterium lactis* have been shown to enhance NK cell activity in both rodent models and human studies [95]. Prebiotic, probiotic, and synbiotic supplementation can enhance treatment tolerance in oncology patients by modulating gut microbiota disrupted by chemotherapy and radiotherapy [96]. Although indirect benefits on treatment response are promising, direct effects on tumor control remain uncertain, underscoring the need for more rigorous and standardized clinical trials [97].

**Figure 2:**
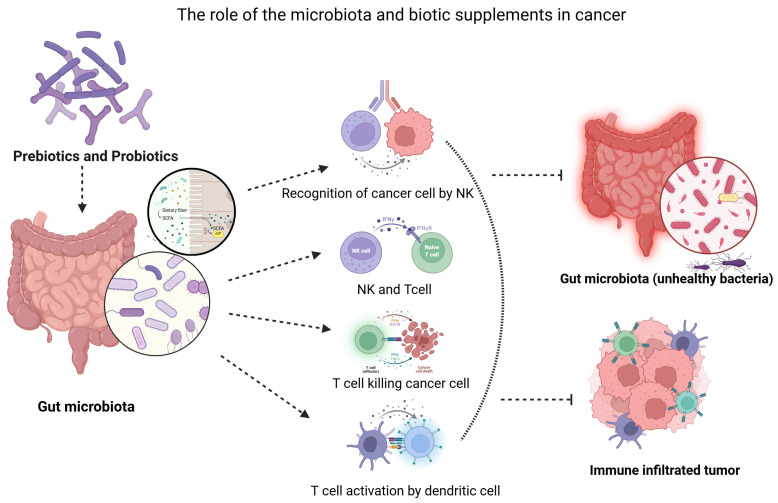
The role of microbiota and biotic supplements in cancer. The figure illustrates the role of the gut microbiota and biotic supplements (prebiotics and probiotics) in cancer immunity. Prebiotics and probiotics positively influence the gut microbiota composition, promoting beneficial bacterial populations. A healthy gut microbiota enhances immune responses against cancer by facilitating the recognition of cancer cells by natural killer (NK) cells and the activation of NK and T cells. These immune cells contribute to the killing of cancer cells and the activation of T cells by dendritic cells, leading to an immune-infiltrated tumor environment that helps control tumor growth. Conversely, an unhealthy gut microbiota with harmful bacteria impairs these immune functions, reducing the body’s ability to fight cancer.

Prebiotics influence cancer development and treatment response through modulation of inflammation, regulation of immune activity, and support of anti-tumor mechanisms. Alteration of gut microbiota and increased short-chain fatty acid production contribute to enhanced immune function, reduced inflammation, and improved gut barrier integrity [99]. A primary mechanism through which probiotics confer protection against cancer development is the modulation of the gastrointestinal microbiome. Probiotics can selectively inhibit the growth of pathogenic bacteria, including *Clostridium difficile*, *Escherichia coli*, and *Fusobacterium nucleatum*, which have been linked to an increased risk of specific cancer types [99]. Although evidence for direct effects on tumour control remains limited, current trials indicate that synbiotics offer significant supportive benefits during cancer treatment and may help maintain a healthier microbiome, which is associated with more favourable clinical outcomes [100] (Figure 2).

Antioxidant-loaded hydrogels combined with microbiome modulators can improve redox homeostasis, decrease inflammation, and modify immunological responses, all of which can increase therapeutic effects [101].

### 3.3. Clinical Relevance and Current Research

Recent advances in next-generation and third-generation sequencing technologies have elucidated the influence of gut microbiota on tumor development and therapeutic response. Distinct microbial compositions differentiate cancer patients from healthy individuals and are associated with immunotherapy outcomes, indicating their potential as biomarkers for predicting treatment efficacy. Interventions, including fecal microbiota transplantation, prebiotics, probiotics, and dietary modifications, have demonstrated potential to enhance the effectiveness of immunotherapy and mitigate treatment-related adverse effects. Collectively, these findings indicate that modulation of gut microbiota may significantly improve cancer treatment outcomes [102]. There is currently little and insufficient data to support the stability and viability of probiotics in hydrogel matrices.

Although probiotics and synbiotics have been shown to have positive effects in preclinical and clinical studies, such as decreased gastrointestinal toxicity and enhanced microbial balance, there is still a lack of clear documentation regarding the survival, metabolic activity, and functional stability of probiotics in hydrogel delivery systems.

Fecal microbiota transplantation (FMT) represents an innovative therapeutic strategy aimed at restoring gut microbiome balance and modulating microbial metabolite levels [103]. Given the bidirectional relationship between gut dysbiosis and cancer development, FMT entails the transfer of healthy microbiota from donors to cancer patients, with the potential to reestablish microbial equilibrium and mitigate adverse symptoms associated with chemotherapy [102]. In a phase 1 trial with patients resistant to anti-PD-1 therapy, the combination of FMT and reinduction of anti-PD-1 resulted in both partial and complete clinical responses. These outcomes were associated with increased gut microbiome diversity, greater intratumoral CD8^+^ T-cell infiltration, and beneficial alterations in immune gene expression, suggesting reprogramming of the immune system. Notably, patients who received FMT from donors with positive responses to immunotherapy experienced better outcomes [104].

Clinical interventions targeting the gut microbiome demonstrate potential for both the prevention and management of colorectal cancer (CRC) [105]. Dietary prebiotics and fiber consistently increase populations of short-chain fatty acid-producing bacteria, a change associated with reduced epithelial proliferation and decreased tumorigenic risk. Probiotic and synbiotic supplementation also promotes beneficial microbial profiles and, in surgical CRC patients, contributes to reduced postoperative infections and complications. Collectively, these findings indicate that microbiome-modulating strategies, including prebiotics, probiotics, and synbiotics, can positively influence CRC-associated microbial imbalances and may enhance treatment outcomes [106].

The composition of the microbiome influences the tumor microenvironment (TME) and modulates treatment responsiveness. In colorectal cancer, elevated intratumoral levels of *Fusobacterium nucleatum* are strongly correlated with disease recurrence and decreased recurrence-free survival [107]. Experimental evidence indicates that *F. nucleatum* reduces the efficacy of 5-fluorouracil by activating anti-apoptotic pathways, including Hippo-YAP–mediated BCL2 upregulation, and by promoting autophagy through TLR4-dependent signaling and microRNA modulation. These observations emphasize the significance of dysbiosis-driven mechanisms in chemoresistance and highlight the therapeutic potential of targeting the gut microbiome to enhance cancer treatment outcomes [108].

Prebiotics have been integrated into chemotherapeutic formulations to improve drug bioavailability and efficacy mediated by the microbiota [109]. For instance, a xylan-based capecitabine complex (SCXN) increased drug bioavailability and concurrently promoted the growth of bacterial taxa such as *Akkermansia* and *Faecalibaculum*, both of which are linked to enhanced treatment outcomes in murine models [108].

The potential to enhance gut health has been established using synbiotics. Pre-clinical and clinical studies suggest that synbiotics have diverse applications in cancer patient management [110]. Research investigated the effect of synbiotics on bacterial translocation and subsequent bacteremia after neoadjuvant treatment for esophageal carcinoma. Twenty patients received synbiotics, while twenty served as controls. The synbiotics group exhibited a lower incidence of grade 3 gastrointestinal toxicity following chemotherapy compared to the control group. These findings suggest that synbiotic administration may reduce bacterial translocation and bacteremia associated with neoadjuvant chemotherapy for esophageal cancer [111].

Prebiotics, probiotics, synbiotics, and postbiotics represent promising microbiome-focused strategies for promoting health and potentially reducing cancer risk. Future research should clarify the mechanisms by which these agents’ function, such as altering microbial populations, generating bioactive compounds, or modulating host responses. This knowledge will be essential for developing effective, personalized cancer prevention and treatment strategies, as well as for designing precision biotics capable of delivering targeted pro-apoptotic factors, tumor suppressor genes, and immune modulators for individualized cancer therapy [112].

### 3.4. Hydrogel-Based Antioxidant Delivery Enhances Microbiome-Mediated Therapeutic Effects

The oxidative state of the tumor microenvironment (TME) is intimately related to the therapeutic effectiveness of microbiome modification in cancer treatment. In addition to tumor growth and genetic instability, elevated reactive oxygen species (ROS) also cause microbial dysbiosis, chronic inflammation, disruption of the epithelial barrier, and compromised immunological response [113]. Under these circumstances, an unfavorable redox landscape may cause microbiome-based treatment techniques to lose their effectiveness. Antioxidant delivery systems based on hydrogel offer a physiologically synergistic and spatially regulated method that can improve microbiome-mediated therapeutic results and restore redox equilibrium [114]. By encouraging pro-inflammatory microbial communities and selectively disadvantaging beneficial commensal species, excessive ROS generation compromises mucosal integrity and fosters dysbiosis [115].

Through reducing oxidative stress directly at the pathological region, injectable or implantable hydrogels’ localized and sustained antioxidant release protects epithelial tight junctions, stabilizes mucosal barriers, and limits oxidative damage to host tissues and commensal microorganisms [116]. The preservation and functional activity of beneficial bacteria are supported by the permissive milieu that hydrogels produce by reestablishing redox balance inside the TME and surrounding mucosal surfaces. The overall consistency of treatment responses is improved and microbiome-driven immune regulation is strengthened by this stabilization [117].

Through a variety of processes, such as dendritic cell activation, T-cell priming, cytokine modulation, and the synthesis of bioactive metabolites such as short-chain fatty acids, the microbiome has anti-cancer effects. On the other hand, long-term oxidative stress causes T-cell fatigue, damages immune cell function, and tilts immunological responses in the direction of immunosuppressive traits (Figure 2).

Antioxidant treatment by hydrogel preserves the ability of dendritic cells to present antigens, lessens T-cell dysfunction brought on by ROS, and restricts the overproduction of pro-inflammatory cytokines. Antioxidant-loaded hydrogels boost the immunomodulatory effects mediated by microbiota and their metabolites, enhancing both local and systemic anti-tumor immunity by preserving immunological competence within an optimal redox environment [116,117].

Through shielding oxygen-sensitive bacterial strains and halting the oxidative destruction of therapeutic molecules, antioxidants also help when hydrogels are designed to co-deliver probiotics, prebiotics, or bioactive substances generated from the microbiome. In order to improve microbial survival, promote colonization, and guarantee continuous bioactive release, the three-dimensional hydrogel matrix provides a protective niche [101]. Antioxidants and microbiome modulators delivered in a coordinated and synchronized manner stimulate the synthesis of immune-regulatory metabolites and encourage microbial metabolic activity. This combination lowers variability in microbiome-based therapies and increases therapeutic stability [118].

In cancer, microbial dysbiosis and oxidative stress create a two-way pathogenic loop. Elevated ROS levels intensify inflammatory signaling pathways, including NF-κB activation, and inflammation further upsets the balance of microbes and strengthens oxidative damage. By lowering tumor-promoting cytokine levels, decreasing oxidative DNA and lipid damage, and attenuating chronic inflammatory signaling, hydrogel-based antioxidant systems break this cycle. The microbiome’s ability to produce anti-inflammatory and immunomodulatory metabolites improves as inflammation subsides and redox equilibrium is restored [119].

The capacity of hydrogel platforms to offer geographically localized, temporally controlled, and stimuli-responsive release is one of its distinguishing features. By delivering antioxidants directly to tumor or mucosal locations, systemic disruption of physiological ROS signaling is reduced [120]. Furthermore, release kinetics can be designed to react to pathogenic triggers like acidic pH, high ROS levels, or tumor-associated enzymes, or they can be synced with microbiome modulators. This accuracy maximizes synergy while minimizing off-target effects by guaranteeing that antioxidant effects are limited to diseased microenvironments where microbiome-mediated therapeutic mechanisms are most active [121].

Oxidative stress in the tumor microenvironment reduces the effectiveness of microbiome-based cancer therapies by promoting dysbiosis and immune dysfunction. Hydrogel-based antioxidant delivery can restore redox balance, protect tissues and microbes, and enhance immune responses [122]. Co-delivery of antioxidants and microbiome modulators may be synergistic, but could also be merely additive or even antagonistic if excessive antioxidant activity interferes with immune signaling or microbial balance. Proper hydrogel design aims to maximize synergy and therapeutic benefit while minimizing unwanted interactions [123].

In summary, by restoring redox equilibrium, stabilizing microbial populations, maintaining immunological function, safeguarding microbiome-targeted therapeutics, and breaking inflammation–oxidative stress feedback loops, antioxidant administration via hydrogels improves microbiome-mediated therapeutic benefits. Hydrogels serve as integrated therapy platforms that mechanistically link microbiome modulation and oxidative stress regulation through controlled and targeted co-delivery, thereby increasing the effectiveness and predictability of cancer management techniques.

### 3.5. Advantages of the Combined Antioxidant–Microbiome Modulation Strategy

Integrating microbiota modification and antioxidant therapy is a rational and mechanistically complimentary approach to cancer treatment. This integrated strategy tackles two interdependent drivers of tumor growth and treatment resistance by concurrently treating oxidative stress and microbial dysbiosis inside the tumor microenvironment (TME) [124].

The restoration of redox equilibrium is one of the main benefits. In addition to encouraging tumor growth and genetic instability, excessive reactive oxygen species (ROS) can compromise immune cell function and upset the delicate balance of microorganisms. By lowering pathogenic ROS levels, antioxidants protect host tissues, maintain the integrity of the epithelial barrier, and provide an environment that is favorable to the survival and efficient operation of beneficial microbial populations [125]. Additionally, the method improves microbial vitality and stability. Antioxidant support increases the survival, colonization, and sustained metabolic activity of many beneficial or therapeutic bacterial strains because they are susceptible to oxidative stress. The generation of immunomodulatory metabolites like short-chain fatty acids is one of the microbiome-mediated therapeutic effects that is more consistent and long-lasting as a result of this stability [126].

Crucially, immunological competence is strengthened by the combination strategy. Redox equilibrium minimizes the chronic overproduction of pro-inflammatory cytokines, maintains the ability of dendritic cells to deliver antigens, and avoids T-cell exhaustion. This leads to improved systemic and local anticancer immune responses when combined with microbiome-driven immune activation. Disrupting the feedback loop between oxidative damage and inflammation is another important benefit. Tumor-promoting signaling and chronic inflammation are sustained by oxidative stress and microbial dysbiosis. By simultaneously stabilizing microbial populations and lowering ROS levels, the combination approach breaks this pathogenic loop and encourages a more controlled inflammatory and immunological environment [127].

Antioxidants also shield bioactive substances produced from the microbiome from oxidative deterioration, increasing their bioavailability and therapeutic effectiveness. Microbiome modulators enhance immunological and metabolic anticancer pathways, whereas antioxidants optimize the microenvironment. Coordinated and spatially controlled co-delivery, when administered through engineered platforms like hydrogels, may have synergistic benefits [128].

In general, the method of combining antioxidants with microbiome modification enables better immune control, better therapeutic stability, protection of beneficial microorganisms and metabolites, and the possibility of synergistic efficacy. This integrative strategy may improve clinical outcomes and therapy predictability in cancer care by targeting both oxidative imbalance and microbial dysbiosis. 

## 4. Hydrogel Platforms for Drug Delivery

### 4.1. Types and Properties of Hydrogels

Conventional drug delivery methods require repeated administration of active compounds to sustain therapeutic concentrations in the body. This approach often reduces patient compliance, diminishes efficacy, and increases the risk of side effects due to elevated dosages [129]. Hydrogels represent a promising platform for diverse applications in drug delivery and regenerative medicine. These materials are soft, three-dimensional (3D) crosslinked polymer networks characterized by high water content [130,131]. Due to their capacity to create an environment conducive to transplanted cell survival and to restore tissue function lost to disease, immunomodulating hydrogels (IMHs) demonstrate significant potential for medical applications, particularly in tissue regeneration, cancer treatment, and inflammation control [132].

Hydrogels are synthesized from a diverse array of natural and synthetic polymers [133]. Synthetic materials provide enhanced structural and mechanical stability. In contrast, natural polymers offer superior biocompatibility, reduced immunogenicity, and improved cell adhesion [134]. Increasingly, hybrid hydrogels that integrate both natural and synthetic components are used to combine the advantages of both material types [135].

Naturally derived hydrogels are generally categorized into three groups: protein-based materials, polysaccharide-based materials, and materials derived from decellularized tissue [136]. These hydrogels are typically composed of proteins and extracellular matrix (ECM) components [137], which confer inherent biocompatibility and bioactivity [138]. As a result, they are potentially suitable for a wide range of biomedical applications due to their ability to promote various cellular functions. Examples of these hydrogels include those derived from collagen, gelatin, elastin, fibrin, and silk fibroin. Elastin, collagen, and fibrin are abundant proteins within the ECM, providing essential strength and elasticity, which makes them promising candidates for tissue engineering and cell culture systems. These proteins are primarily sourced from animals; for instance, collagen is typically extracted from porcine tissue or murine tails, while fibroin is obtained from insects [139].

Synthetic polymers are considered advantageous for hydrogel synthesis due to their highly controllable physical and chemical properties compared to natural polymers. These polymers can be engineered with long-chain structures and high molecular weights. However, synthetic polymer hydrogels generally exhibit lower biological activity than their natural counterparts [140]. Various synthesis methods are available for synthetic polymer hydrogels, including the use of polymerizable vinyl monomers and chemical crosslinking of polymers. Common synthetic polymers utilized in hydrogel synthesis include poly(vinyl alcohol) (PVA), poly(ethylene glycol) (PEG), poly(ethylene oxide) (PEO), poly(2-hydroxyethyl methacrylate) (PHEMA), poly(acrylic acid) (PAA), and poly(acrylamide) (PAAm) [140,141].

A third category includes semi-synthetic hydrogels, such as gelatin methacryloyl hydrogels, which are gelatin-based materials functionalized with synthetic methacryloyl groups [142].

Hydrogels are classified according to composition and structure. Based on polymeric composition, they include homopolymeric hydrogels synthesized from a single monomer, copolymeric hydrogels formed from multiple monomers, and interpenetrating polymer networks (IPNs) composed of two overlapping polymer networks [143]. Semi-IPNs contain only one cross-linked component. Structurally, hydrogels are categorized as amorphous, semicrystalline, or crystalline. Classification by cross-linking distinguishes chemically cross-linked hydrogels, which possess permanent bonds, from physically cross-linked hydrogels, which rely on reversible interactions such as hydrogen bonding or ionic forces. In terms of physical form, hydrogels may exist as matrices, films, or microspheres. Classification by network charge includes non-ionic, ionic, amphoteric, and zwitterionic hydrogels, depending on the presence and type of charged groups within the polymer chains [144].

Hydrogels possess unique properties that enable their use in diverse applications. Specifically, their water-rich, 3D network structure facilitates adequate diffusivity and creates an environment that closely mimics physiological conditions for cell and tissue metabolism. These characteristics render hydrogels promising platforms for biomedical applications, including drug delivery, cell culture, and tissue regeneration [145].

Hydrogels have been widely utilized as 3D models for various diseases, including tumor models, tissue fibrosis models, corneal disease models, nerve disease models, and inflammatory bowel disease, for the investigation of pathogenesis and high-throughput drug screening. Owing to their ability to mimic the in vivo tissue stroma matrix, hydrogels are well-suited for cell encapsulation and expansion both in vitro and in vivo [146], thereby facilitating efficient tissue regeneration and cancer therapy. For example, various cell types, including stem cells, islet cells, hepatocytes, and endothelial cells (ECs), encapsulated within hydrogels, can proliferate while maintaining their functional characteristics in vitro [147].

Though hydrogels may have limited mechanical strength and stability, natural hydrogels like hyaluronic acid, chitosan, and alginate offer exceptional biocompatibility, biodegradability, and innate bioactivity [148]. Synthetic hydrogels, such as those based on polyethylene glycol (PEG), poly(vinyl alcohol) (PVA), and PLGA, offer controlled degradation, repeatability, and adjustable mechanical properties; however, they might not be bioactive [149]. The advantages of both natural and synthetic polymers are combined in hybrid hydrogels, which improve mechanical resilience, allow for controlled release, and shield delicate bioactives. This comparative analysis emphasizes the suitability of each hydrogel type for targeted, sustained, and microbiome-friendly delivery in cancer therapy, providing a more critical and informative perspective than a simple listing of examples.

*The appropriateness of various hydrogel delivery methods for cancer treatment that targets the microbiota.*

Injectable hydrogels minimize systemic exposure and allow for prolonged release by localized distribution to gastrointestinal regions or tumor locations [150]. Oral hydrogels are especially well-suited for regulating gut flora and shielding postbiotics or probiotics from stomach breakdown. Long-term, site-specific release is provided by implantable hydrogels, which may be useful in applications near tumors or after surgery [151]. Topical hydrogels have limitations for systemic microbiome-targeted therapy, although they may be useful for skin-associated microbiome modification [117]. In conclusion, we present how the selection of a hydrogel system may be adapted to the therapeutic target and delivery needs in microbiome-based cancer therapies by critically analyzing different routes of administration.

### 4.2. Synthesis and Functionalization Methods

Hydrogel synthesis is a critical process in the development of novel structures with advantageous properties for drug delivery applications. The structure of hydrogels is determined by the hydration of hydrophilic groups and polymer domains. These groups and their interconnected chains form 3D networks through crosslinking, which prevents dissolution in the aqueous phase [152]. Hydrogels can be categorized according to their cross-linking mechanisms as physically cross-linked, chemically cross-linked, or hybrid physically and chemically cross-linked types [153].

Physically cross-linked hydrogels rely on non-covalent interactions such as hydrogen bonding, ionic forces, and hydrophobic interactions [154]. These reversible cross-links confer high sensitivity to environmental cues such as pH, temperature, and ionic strength, and enable properties including self-healing [153]. The absence of toxic chemical cross-linking agents in the gelation process enhances their safety for clinical applications, and preparation under mild conditions makes them suitable for sensitive drug loading. However, the transient nature of these cross-links results in a short lifespan, typically ranging from several days to one month in physiological media, which restricts their use to applications requiring short-term drug release [155].

Chemically cross-linked hydrogels are synthesized by forming permanent covalent bonds between polymer chains, using various chemically active motifs and methods such as free radical polymerization, carbodi-imide chemistry, or highly selective click chemistry. This strategy enhances matrix stabilization and allows for greater control over gel formation and final structure compared to physical gels [156]. Techniques such as enzymatic crosslinking, which utilize biocompatible enzymes under mild conditions, are especially preferred for producing non-cytotoxic hydrogels suitable for advanced applications in tissue engineering, drug delivery, and regenerative medicine [157]. Additionally, high-energy radiation methods can be employed to efficiently initiate polymerization and precisely tailor the hydrogel network [156].

The synthesis method determines the fundamental stability and porosity of the hydrogel network, as established by physical or covalent cross-linking [140]. Subsequent functionalization strategies are crucial for incorporating specific biological, chemical, or physical motifs that facilitate controlled drug loading, triggered release, and targeted delivery.

Mechanochemistry represents a promising yet underexplored strategy for hydrogel functionalization, utilizing applied force to induce dynamic changes within the polymer network. This approach integrates force-responsive molecular linkages, known as mechanophores, to regulate functions such as optical property modulation, mechanical behavior, and, importantly, the immobilization and release of molecules or controlled polymer degradation [158].

However, the application of mechanochemistry to hydrogels presents several significant challenges. Mechanophores are frequently hydrophobic, which limits their compatibility with aqueous hydrogel environments and water can alter mechanochemical activity [159]. Moreover, hydrogels are often brittle, which prevents the generation of sufficient force required for activation. Recent advances have addressed these challenges by developing multi-responsive hydrogels that incorporate mechanophores, such as anthracene–maleimide, alongside other responsive elements, including ultraviolet light-sensitive motifs or disulfide bonds. This design enables independent or combined activation of distinct responses, such as force-triggered degradation or ultraviolet-triggered cell attachment. These systems offer spatial control over cell adhesion and are highly versatile for drug delivery applications [160].

An alternative and highly versatile functionalization strategy is the development of Nanoparticle-Hydrogel Hybrid Systems. Nanoparticle (NP)-based drug delivery carriers, including lipid NPs (LNPs) [161], polymeric NPs [162], silica, carbon-based NPs, and inorganic NPs such as silver, gold, titanium, metal oxides, and metal alloys, have been developed for a range of biomedical applications, including cancer therapy [163]. NP-based drug delivery systems offer several advantages, such as high drug loading capacity, protection of drugs from premature release, responsiveness to external stimuli, the ability to cross biological barriers, and targeted delivery to specific cells or organs through tunable particle size and structure [164]. These hybrid materials further enhance stability, for example, hydrogel coatings protect LNP-loaded drugs from the acidic stomach environment to enable targeted colon delivery, improve drug loading, and introduce multifunctional capabilities. Embedding magnetic NPs, for instance, enables accelerated drug release in response to an external magnetic field or facilitates the hyperthermia effect, thereby improving therapeutic efficacy and tissue penetration [163].

### 4.3. Advantages for Targeted Cancer Therapy

Traditional cancer treatments include surgical removal of tumors, systemic administration of anti-cancer drugs through chemotherapy, and the use of ionising radiation or charged particle beams in radiotherapy [165]. Biocompatible hydrogels with multinetwork and porous structures have been extensively utilized in biomedical applications, including bone tissue regeneration, wound healing, antimicrobial therapy, biosensing, and cancer treatment. Hydrogels offer significant advantages as novel drug carriers, with the potential to transform cancer treatment strategies. Notably, hydrogels provide robust drug delivery capabilities and enable precise control over drug release [166].

A significant side effect of cancer treatment is the inadvertent damage to normal tissue. The removal or clearance of carcinoma tissue inevitably results in tissue loss, which can diminish patients’ quality of life [167]. In recent years, a two-stage approach has been proposed: first, eliminating tumor tissue, and second, providing an appropriate scaffold or stimulating factor to facilitate tissue regeneration. Hydrogels are well-suited for this application due to their sensitivity to various biological signals, controlled drug release capabilities [168], and ability to promote histogenesis [169]. Following the elimination of abnormal tissue, surrounding normal host stem cells can migrate to the defect area and form new tissues through proliferation and differentiation. Several studies have successfully implemented this strategy. Furthermore, as cancer therapy moves toward more precise and individualized approaches, hydrogels are anticipated to play a promising role in the combined treatment of localized tumor therapy and tissue engineering [170].

Multiple hydrogel platforms are currently undergoing clinical trials, primarily employing thermoresponsive polymers for localized and sustained drug delivery in oncology [171]. For instance, ReGel, a Poloxamer-based system combined with Paclitaxel, has been evaluated for non-muscle invasive bladder cancer and pancreatic cancer, demonstrating reduced systemic toxicity and elevated local drug concentrations in phase II trials. Another example is a thermosensitive PEG-co-poly(glycolic acid) formulation (Encapson), which serves as a long-acting local anesthetic by gradually releasing Bupivacaine for post-operative pain relief, resulting in extended analgesia lasting up to three days [170].

Hydrogels are increasingly employed to develop sophisticated 3D tumor models that incorporate the cellular diversity characteristic of native tumor tissues, including tumor cells, fibroblasts, and macrophages [172]. This approach enhances the predictive accuracy of drug testing because cell–cell interactions play a critical role in modulating therapeutic responses [173]. For example, enzymatically cross-linked silk fibroin hydrogels have facilitated the coculture of breast cancer cells with tumor-associated fibroblasts (TAFs), demonstrating that TAFs can induce chemoresistance in cancer cells, a phenotype frequently observed in native tumors. In a similar manner, alginate cryogels supporting organoids cocultured with monocyte-induced macrophages, which simulate the immune microenvironment, have been shown to promote increased tumor growth and more aggressive phenotypes [174].

In addition to basic coculture approaches, advanced methodologies integrate hydrogels with microfluidic devices, such as 3D bioprinted scaffolds, to replicate complex tumor heterogeneity and facilitate the study of cell migration [175]. Although these multi-cellular hydrogel systems hold significant promise for identifying native-like cancer cell phenotypes, future research should prioritize validating their effectiveness for precision medicine and therapeutic testing, particularly in relation to the substantial drug resistance characteristic of native tumors [176].

### 4.4. The Relationship Between Probiotics and Antioxidants in Cancer with Emphasis on Signaling Pathways in the Digestive Tract

Probiotics and antioxidants work together to influence important signaling pathways in the gastrointestinal tract, which is the basis for their interaction in cancer prevention and progression. These pathways control immunological responses, oxidative stress, inflammation, and epithelial integrity, all of which are directly related to the development and spread of tumors. Probiotics and antioxidants have parallel mechanistic effects on the gut’s local microenvironment as well as systemic signaling networks linked to cancer development [13].

The main ways that probiotics influence the gut microbiota and the synthesis of bioactive metabolites are through their impact on signaling regulation linked to cancer. The short-chain fatty acids (SCFAs), especially butyrate, are among the most significant metabolites produced by probiotic bacteria. In addition to providing colonocytes with energy, butyrate inhibits histone deacetylases (HDACs), which has epigenetic effects [177]. Chromatin structure and gene expression are altered by HDAC inhibition, which suppresses genes linked to unchecked proliferation and increases the transcription of genes involved in death and cell cycle regulation. Because it reverses the dysregulated gene expression patterns frequently seen in tumor cells, this epigenetic modification is important in the biology of cancer [178].

Furthermore, butyrate stimulates signaling pathways like AMPK, which controls cellular energy balance and has the ability to block the mTOR pathway, which is essential for cell division and growth. Probiotics affect inflammatory signaling pathways in addition to metabolite synthesis. Chronic inflammation is known to play a role in the development of cancer, especially in the gastrointestinal tract, where inflammatory mediators foster a microenvironment that damages DNA and encourages the growth of tumors. The transcription factor NF-κB, which controls the expression of pro-inflammatory cytokines like TNF-α and IL-6, is less activated while taking probiotics. Probiotics reduce inflammatory signaling cascades that might otherwise encourage cellular survival and proliferation by inhibiting NF-κB signaling. The improvement of intestinal barrier function supports this anti-inflammatory action [179].

Through strengthening tight junction proteins, probiotics lower intestinal permeability and stop microbial products like lipopolysaccharides (LPS) from moving into the bloodstream. Since LPS is a strong immune signaling pathway activator, systemic inflammation and related pro-tumorigenic signals are limited by its decreased translocation [180].

Via their modulation of oxidative stress and redox-sensitive signaling pathways, antioxidants interact with these activities. Reactive oxygen species (ROS) serve as signaling molecules in addition to being byproducts of cellular metabolism. ROS are involved in cell signaling mechanisms at physiological levels, while excessive ROS production causes oxidative damage to proteins, lipids, and DNA. Since DNA damage can lead to mutations that develop tumors, it represents an important stage in the carcinogenesis process [125].

Additionally, signaling pathways that control inflammation and cell survival are impacted by oxidative stress. Both the NF-κB and MAPK pathways, which are implicated in proliferative and inflammatory responses, are activated by ROS. Indirectly, antioxidants lessen the activation of these pathways by lowering ROS levels. This signaling modification limits the creation of inflammatory mediators and lowers the proliferative signals that fuel tumor growth, among other downstream impacts on gene expression. Additionally, by modifying immune cell function, antioxidants can affect the tumor microenvironment. Antioxidants promote immunological activity by maintaining the functionality of immune cells and improving their ability to identify and eradicate aberrant cells, while oxidative stress lowers immune surveillance mechanisms [181]. By taking into account the combined effects of probiotics and antioxidants on digestive system signaling, the interaction between the two becomes clear.

Probiotics change the composition and function of the gut microbiota, which in turn affects inflammation and oxidative stress. The production of metabolites such as SCFAs by a healthy microbiota enhances the expression of antioxidant enzymes, supporting redox balance and immunological control. Conversely, dysbiosis raises inflammation and oxidative stress, which may aid in the development of cancer. Probiotics support a more balanced and protective gut environment by restoring bacteria diversity and combating dysbiosis [182].

Together, probiotics and antioxidants can lower oxidative and inflammatory signals linked to cancer. Antioxidants specifically target redox-sensitive pathways, whereas probiotics alter the gut flora and immunological responses. By lowering oxidative stress, they prevent pro-inflammatory signals like NF-κB from being activated, which aids in suppressing circumstances that promote the growth of tumors [183]. In the tumor microenvironment, their joint activity addresses both metabolic processes and microbial imbalance, demonstrating a synergistic protective effect.

Since the digestive tract interacts with the immunological, metabolic, and endocrine systems, gut signaling connects local intestinal processes with the risk of systemic cancer. Through lowering intestinal inflammation and enhancing barrier integrity, probiotics and antioxidants modulate this network and reduce systemic exposure to inflammatory mediators. Beneficial bacteria-produced SCFAs also have metabolic and epigenetic effects that can control cellular activity outside of the gut. These processes demonstrate how changes in gastrointestinal signals may have an effect on cancer risk at the local and systemic levels [184].

In conclusion, the relationship between microbiome modulators and antioxidants in cancer stems from their capacity to alter signaling pathways inside the digestive system. Antioxidants lessen oxidative stress and its effects on redox-sensitive pathways, whereas probiotics affect the makeup of microorganisms and generate compounds that control epigenetic and inflammatory signaling. Via improving redox balance, lowering inflammation, and promoting genomic integrity, these processes work in tandem.

**Figure 3:**
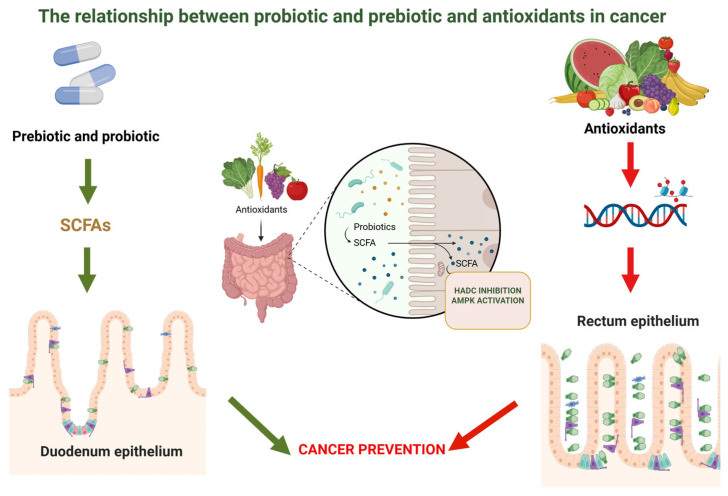
The relationship between probiotic and prebiotic and antioxidants in cancer. Prebiotics support beneficial gut bacteria and help metabolic and immune balance. Probiotics generate short-chain fatty acids, especially butyrate, which nourish colon cells and influence gene regulation. Butyrate helps control cell growth and promotes removal of damaged cells. Probiotics also reinforce the gut barrier, reducing permeability and inflammatory triggers. A stronger intestinal barrier limits exposure to harmful signals. Antioxidants dampen inflammatory pathways and decrease conditions linked to tumor development. They also protect immune cells, improving host defense. Together, probiotics and antioxidants reduce oxidative stress and inflammation, both major contributors to cancer risk. Short-chain fatty acids support gene mechanisms that maintain controlled proliferation. Overall, microbiota modulation and antioxidant protection enhance tissue homeostasis and genomic stability.

Combining antioxidants and microbiota modulators in a single hydrogel substrate is not a straightforward additive method, but rather a logical and mechanistically supported therapeutic strategy. Microbiota imbalance, inflammation, oxidative stress, and epithelial barrier dysfunction are all biologically related processes that contribute to the development of disease. Merely focusing on one of these elements frequently yields minimal or temporary advantages. Antioxidant and microbiome modulator administration, on the other hand, concurrently treats microbial dysbiosis, inflammatory signaling, redox imbalance, and barrier integrity (Figure 3).

Through guaranteeing localized, regulated, and prolonged release, safeguarding delicate bioactive chemicals, and improving their stability and bioavailability, the hydrogel matrix reinforces this approach even more. While lowering systemic exposure and possible side effects, this temporal and geographical control improves therapeutic accuracy.

Furthermore, the simultaneous regulation of oxidative stress and microbiota composition might affect important cellular processes related to immune surveillance, apoptosis, proliferation, and genomic stability, hence reducing the conditions that promote tumor growth and chronic inflammation.

Consequently, a multifunctional hydrogel platform that co-delivers microbiota modulators and antioxidants offers higher translational potential, improved treatment efficiency, and synergistic biological benefits. In addressing illness complexity through coordinated, multi-target intervention rather than discrete therapy modalities, this strategy is consistent with contemporary precision medicine approaches.

## 5. Incorporation of Natural Antioxidants into Hydrogels

### 5.1. Techniques for Embedding Antioxidants

Natural antioxidants such as catechins, flavonoids, carotenoids and polysaccharides derivatives can be physically entrapped or chemically conjugated within hydrogel matrices. Physical encapsulation relies on non-covalent interactions, including hydrogen bonding, electrostatic interactions, and hydrophobic forces, allowing for mild fabrication conditions that preserve the biological activity of sensitive antioxidant molecules. In contrast, chemical conjugation strategies can provide enhanced stability and prolonged retention within the hydrogel network, although careful design is required to avoid compromising antioxidant functionality.

A critical aspect of designing effective antioxidant-loaded hydrogels is the method used to incorporate the active compounds into the polymer network. Various embedding techniques ranging from simple physical entrapment to advanced chemical conjugation and stimuli-responsive strategies allow precise control over loading efficiency, stability, release kinetics, and therapeutic performance.

The choice of base polymer critically influences the mechanical properties and functional behavior of antioxidant delivery systems. Comparative studies demonstrate that N-carboxyethyl chitosan-based hydrogels exhibit greater intrinsic gel strength than hyaluronic acid (HA) formulations due to stronger polymer–polymer interactions. This enhanced mechanical integrity is directly associated with prolonged retention of embedded antioxidants and slower release rates. As a result, the biological activity and residence time of resveratrol can be tailored by rationally tuning the hydrogel’s structural properties [185,186,187].

Stimuli-responsive hydrogels further advance antioxidant delivery by enabling environment-triggered release. These systems respond dynamically to pathological cues such as the mildly acidic conditions (pH 6.5–7.0) characteristic of tumor microenvironments or inflamed tissues [188,189,190,191]. Temperature-responsive hydrogels enhance the practicality of localized delivery. Materials such as Pluronic F127 combined with chitosan undergo rapid sol–gel transitions at physiological temperature (37 °C), allowing in situ gelation after injection. This property ensures site-specific retention and sustained antioxidant release without invasive implantation procedures [191,192].

Hydrogels sensitive to ROS represent a more specialized embedding strategy designed to exploit the elevated oxidative stress present in diseased tissues [188,193]. In addition to passive encapsulation, polyphenolic antioxidants can actively participate in hydrogel network formation. Owing to their aromatic rings and phenolic hydroxyl groups, polyphenols can act as physical crosslinkers through hydrogen bonding, often imparting pH-responsive behavior. A particularly robust strategy involves metal–phenolic coordination, where catechol-containing polyphenols bind metal ions such as Fe^3+^ or Cu^2+^, forming dynamic networks with self-healing and mechanically reinforced properties [194,195,196].

When greater structural stability is required, chemical crosslinking strategies are employed. Covalent bonding between phenolic groups and polymer backbones ensures permanent antioxidant integration, using methods such as enzyme-mediated crosslinking, free-radical polymerization, or click chemistry [197,198].

1. EGCG has been successfully incorporated into various hydrogel-based systems to improve its stability, bioavailability, and controlled release. For instance, EGCG-loaded mucoadhesive NanoCubogels (nanocubes embedded in hydrogel) demonstrated prolonged local release and enhanced permeation for potential oral applications. Similarly, co-encapsulation of EGCG with probiotics in alginate-whey protein isolate hydrogel beads protected both components during storage and gastrointestinal transit, enabling sustained release [199,200].

2. Lycopene, a potent carotenoid antioxidant, benefits from hydrogel encapsulation to overcome its hydrophobicity and instability. Lycopene-loaded emulsions stabilized within alginate hydrogel beads exhibited improved storage stability and controlled release during in vitro digestion, with higher bioaccessibility compared to non-encapsulated forms [200,201].

3. β-Glucans, recognized for their intrinsic antioxidant properties alongside immunomodulatory effects, are frequently used as the hydrogel matrix itself rather than solely as embedded guests. Oat β-glucan incorporated into konjac glucomannan hydrogels enhanced gel strength, water-holding capacity, and thermal stability through hydrogen bonding. Oxidized β-glucan hydrogels crosslinked with antimicrobial peptides have also shown multifunctional wound-healing capabilities, leveraging β-glucan’s antioxidative and anti-inflammatory roles [202,203] (Table 4).

### 5.2. Stability, Release Kinetics, and Bioactivity Maintenance

The influence of fabrication and crosslinking conditions on bioactivity requires deeper analysis. The stability of polyphenols is inherently fragile, determined by their molecular structure and susceptibility to environmental factors such as oxygen, temperature, and pH. These factors make them highly vulnerable to degradation during manufacturing and storage [204].

Therefore, careful adjustment of the factors involved in hydrogel production, such as the crosslinking density, polymer composition, and processing conditions, is crucial. Future studies should systematically assess the effects of these factors on polyphenol stability, preservation of bioactivity, and controlled release profiles. This will give researchers a logical foundation for designing hydrogel matrices with the highest possible therapeutic efficacy and shelf life.

Hydrogel encapsulation serves as a crucial engineering solution to this issue, protecting the structural integrity of polyphenols and preventing the loss of bioactivity caused by external factors. This protective environment is necessary to improve the bioavailability and overall shelf-life of the polyphenolic compounds [204,205].

To accurately quantify the stability of encapsulated compounds, researchers utilize quantitative metrics that go beyond simple concentration measurement. More reliable indices include the calculation of the early degradation time and the half-degradation time [206,207].

Furthermore, modeling the reaction kinetics of the degradation process is essential for predicting and controlling the performance of the antioxidant over the lifespan of the hydrogel. Characterizing stability also includes monitoring the compound’s antioxidant potential, as a loss in chemical structure directly correlates with a reduction in therapeutic function [208,209].

The release of drug solutes from hydrogel networks is often modeled using Fickian diffusion theory. This process can be described by a partial differential equation (PDE) for concentration changes over time and space. As the hydrogel degrades, its internal mesh size increases, leading to higher solute diffusivity and faster release [210].

To analyze drug release from polymer matrices involving multiple mechanisms, researchers commonly use the semi-empirical Korsmeyer–Peppas power law model. The key diffusional exponent from this model reveals the dominant release mechanism. A value of 0.5 indicates Fickian diffusion controlled by polymer mesh size. In contrast, 1.0 suggests surface erosion dominance. Intermediate values describe anomalous transport combining diffusion with matrix relaxation or erosion [211,212].

The overall design of the hydrogel, particularly its crosslinking density and mechanical strength, primarily controls drug release kinetics. Higher strength effectively prolongs active agent retention, modulating therapeutic effects [213,214]. Precise engineering of the hydrogel structure enables spatiotemporal control over delivery. While sustained release often maintains local concentrations for extended periods, advanced designs can also support rapid deployment. For instance, stimuli-responsive systems trigger high burst release upon detecting specific environmental cues [188].

The bioactivity of antioxidants incorporated into hydrogels is typically assessed in vitro through standardized spectrophotometric assays. These tests measure the ability of released antioxidant compounds to scavenge or neutralize free radicals, thereby confirming that the encapsulation and release processes preserve the compounds’ functional properties [215,216]. Commonly employed methods include the DPPH (2,2-diphenyl-1-picrylhydrazyl) radical scavenging assay and the FRAP (Ferric Reducing Antioxidant Power) assay. Additional well-established techniques are the ABTS (2,2′-azinobis(3-ethylbenzothiazoline-6-sulfonic acid) radical scavenging assay and the ORAC (Oxygen Radical Absorbance Capacity) assay [216]. These assays provide reliable and reproducible means to evaluate antioxidant performance. For instance, dynamically cross-linked chitosan-based hydrogels have demonstrated markedly enhanced radical scavenging activity relative to native chitosan when tested using the DPPH assay, highlighting the potential benefits of advanced hydrogel formulations in preserving and delivering bioactive antioxidants [217,218].

Translational relevance is established by moving from in vitro assays to complex biological models. Ex vivo studies often confirm biocompatibility, such as complex alginate hydrogels loaded with curcumin-synthesized cerium oxide nanoparticles (Cur-CeO) showing good viability for L929 fibroblasts [219,220].

For clinical applications, in vivo assessment is paramount. Studies utilizing Cur-CeO hydrogel nanocomposites in rat models demonstrated efficacy in accelerating circular deep wound repair. The underlying therapeutic mechanism involves the reduction in oxidative stress and local inflammation, which promotes faster tissue recovery by increasing keratinocyte and fibroblast migration onto the wound bed [220,221].

It is still difficult to integrate hydrogel-based delivery systems and microbiome-modulating techniques into standard clinical practice, even with the encouraging preclinical and early clinical data. To guarantee safety and effectiveness, regulatory frameworks demand strict material composition characterisation, repeatable production procedures, long-term stability, and batch-to-batch uniformity. Furthermore, because these systems are multifunctional and frequently combine biomaterials, bioactive chemicals, and living microbes, they provide difficult regulatory classification problems because they can be classified as drugs, devices, or combination products. The development of standardized potency and quality-control assays, scalable manufacturing, and sterilization without compromising bioactivity are additional translational hurdles. To promote clinical adoption, it will be crucial to address these translational and regulatory obstacles by establishing standardized evaluation procedures and interacting with regulatory bodies early on.

### 5.3. Examples from Recent Studies

Hydrogels formed rapidly under physiological conditions, reaching full gelation within 30 min. Sustained release kinetics were quantified by high-performance liquid chromatography (HPLC) through long-term incubation in phosphate-buffered saline (PBS) and periodic collection of supernatants over several weeks [222].

Incorporating nanoparticles into resveratrol (RSV) hydrogels enhances therapeutic efficacy. Graphene oxide (GO) enables chemo-photothermal therapy, suppressing tumor growth by over 90% in models.

ROS-sensitive hydrogels with MXene nanosheets improve drug penetration by over 3-fold in neuro-oncology models, demonstrating synergistic benefits for targeting difficult sites [188].

Lignin, a natural polyphenol, is valued for its inherent antioxidant and antibacterial capabilities, alongside its biodegradability. A sophisticated approach involves developing injectable, self-assembling hydrogels by combining phenolated lignin nanoparticles (PheLigNPs) with biopolymers such as thiolated hyaluronic acid (HA-SH) and silk fibroin. The PheLigNPs were synthesized via a sono-enzymatic platform involving the laccase-catalyzed oxidation of lignin and grafting with tannic acid [223].

The resulting hydrogels are designed as multifunctional platforms, targeting the complexities of chronic wounds by addressing both bacterial contamination and oxidative enzymes. The incorporated NPs fulfill both structural and functional roles, conferring shear-thinning properties necessary for injectability and maintaining high intrinsic antimicrobial activity by inducing high levels of reactive oxygen species in contacted bacteria [224,225]. Tannic acid (TA) has been effectively utilized in copolymerization schemes, specifically in systems combining acrylic acid (AAc) and 1-vinylimidazole (VI), to construct advanced polyphenol-enhanced wet adhesion hydrogels. This method results in materials that offer synergistic benefits [205].

The incorporation of TA not only provides intrinsic ROS scavenging capacity but also significantly enhances the hydrogel’s mechanical performance. Quantitative measurements via peel and lap-shear tests demonstrated that the material’s toughness and wet tissue adhesion energy increased directly with the concentration of the integrated TA. These enhanced properties are essential for translational applications, particularly in managing infected diabetic wounds where robust, adherent dressings are crucial for promoting localized healing [226,227,228].

EGCG has been incorporated into glucose-responsive GelMA-CPBA (Gelatin methacryloyl functionalized with 3-(Carboxyphenyl)boronic acid) hydrogels through reversible boronic ester interactions with its catechol groups. These photo-crosslinked hydrogels exhibit glucose-triggered EGCG release, enabling adaptive antioxidant delivery in diabetic wound environments. Elevated glucose levels accelerate EGCG liberation, resulting in efficient ROS scavenging, enhanced angiogenesis, and improved collagen remodeling via upregulation of hypoxia-inducible factor-1α (HIF-1α). Collectively, these properties lead to significantly faster wound closure compared with non-responsive hydrogel systems [229].

Lycopene has been nanoemulsified and incorporated into GelMA/alginate hydrogels using UV-mediated crosslinking. This strategy stabilizes the polyene structure of lycopene and prolongs its antioxidant activity within diabetic wound sites. Sustained lycopene release from the hydrogel matrix has been shown to enhance vascular endothelial growth factor (VEGF) expression, promote re-epithelialization, and support dense granulation tissue formation over a 21-day healing period in streptozotocin-induced diabetic models [230].

β-Glucans has been integrated into hyaluronic acid (HA)-based granular hydrogels to address oxidative stress in osteoarthritic cartilage defects. These hydrogels exploit β-glucan–mediated activation of the Dectin-1 receptor, leading to amplification of the Nrf2/HO-1 antioxidant pathway and efficient superoxide neutralization. In addition to their shear-thinning and injectable properties, β-glucan–laden hydrogels suppress TLR-2/NF-κB-associated senescence signaling and promote chondrocyte proliferation, resulting in superior extracellular matrix remodeling compared to commercial HA viscosupplements [231,232].

## 6. Encapsulation of Microbiome Modulators in Hydrogels

### 6.1. Strategies for Probiotic and Prebiotic Encapsulation

Hydrogel encapsulation presents a viable way to get around these restrictions by offering protection and controlled release. Hydrogels are three-dimensional networks of polymers that can hold a lot of water, simulating the conditions found in natural tissues. They are perfect for encasing probiotics and prebiotics because of their permeability, biocompatibility, and adjustable mechanical characteristics. Hydrogels can be designed to release their payload in particular areas of the gut and shield delicate microbiome modulators from severe gastrointestinal conditions [63]. Probiotics are live, health-promoting beneficial bacteria that, when taken in sufficient quantities, can improve health. Encapsulation in hydrogels has been thoroughly investigated to preserve their viability during processing, storage, and passage through the gastrointestinal tract [233].

Alginate, chitosan, carrageenan, and gelatin are typical hydrogel ingredients. Alginate’s moderate gelation process by ionic crosslinking with calcium ions, which maintains cell viability, makes it very well-liked. Alginate beads are frequently coated with chitosan to increase their mechanical strength and offer defense against stomach acid [233].

Probiotic-containing hydrogel beads or microcapsules are created using microencapsulation methods such as extrusion, emulsion, and spray drying. By shielding the bacteria from bile salts and acidic pH, these beads improve colonization and survival rates in the intestines. In order to ensure targeted distribution, recent developments include the use of stimuli-responsive hydrogels, which release probiotics in reaction to particular pH shifts or enzyme activity in the gut [234]. Prebiotics are indigestible fibers or substances that specifically promote the growth of good gut flora. The purpose of their encapsulation is to regulate their release and shield them from early fermentation or deterioration.

Polysaccharides like pectin or inulin, which act as both carriers and prebiotic substrates, are frequently incorporated into hydrogels used for prebiotic encapsulation. Probiotic-like methods, such as ionic gelation and coacervation, can be used to encapsulate hydrogels.

Prebiotics and probiotics are co-encapsulated in a novel method known as “synbiotic delivery,” which provides both the beneficial microorganisms and their food source in a single formulation. This approach supports a balanced gut flora and increases probiotic survival and activity [235]. Optimizing hydrogel formulations to balance protection, release kinetics, and biocompatibility is still difficult despite tremendous improvements. Another challenge is increasing output while preserving the viability of probiotics. Future studies will concentrate on creating intelligent hydrogels that can be released on demand in response to gut microenvironmental cues like pH or microbial enzymes [236].

In order to sum up, encasing microbiome modulators in hydrogels is a potentially effective way to increase the effectiveness of prebiotics and probiotics. More potent microbiome-targeted treatments and functional meals will be made possible by ongoing advancements in material science and delivery systems.

### 6.2. Encapsulation Feasibility

Building on the methods for hydrogel-based probiotic and prebiotic encapsulation covered in the preceding section, material characteristics and experimental data provide compelling evidence for the viability of these tactics. Hydrogels are especially well-suited for shielding delicate microbiome modulators because of their three-dimensional polymeric networks and high water content, which create an environment that resembles natural tissues [237]. Their permeability and biocompatibility, as previously mentioned, enable controlled release and defense against gastrointestinal stresses, hence resolving important drawbacks of probiotic and prebiotic direct delivery.

The methods described, including the application of hydrogels based on alginate and microencapsulation techniques, show realistic routes to develop effective delivery systems. Because alginate gels under mild conditions through ionic crosslinking with calcium ions, microbial viability is maintained throughout the encapsulating process [237,238]. Additionally, the use of chitosan coatings strengthens the protective role of hydrogel matrices by improving mechanical stability and adding resistance to acidic conditions. These results support the previously described controlled-release techniques, highlighting the viability of targeted administration in the gastrointestinal system. Furthermore, by allowing release in reaction to particular environmental triggers like pH changes or enzyme activity, the creation of stimuli-responsive hydrogels enhances the case for feasibility [239].

Prebiotics and probiotics should be delivered exactly where they work best, minimizing premature release and optimizing therapeutic action. A major breakthrough in hydrogel design, the incorporation of such intelligent materials facilitates the use of microbiome-targeted treatments [240]. Additionally, prebiotic encapsulation shows great viability since polysaccharides such as pectin and inulin are compatible with hydrogel systems. In addition to acting as carriers, these substances also function as substrates that encourage the development of advantageous gut microbiota, supporting the previously mentioned synbiotic delivery methods. The simultaneous administration of probiotics and their nutritional support is made possible by these compounds’ dual functionality, which further improves the usefulness of hydrogel-based systems [233].

The overall viability of hydrogel encapsulation is well supported by current research and technological advancements, despite unresolved issues including optimizing release kinetics and scaling production without sacrificing viability [241]. For functional meals and therapeutic interventions to advance in real-world applications, these issues must be resolved through material innovation and process optimization [242]. In conclusion, the viability of encapsulating using hydrogel is a logical progression of the tactics that were previously addressed. The stability and effectiveness of probiotics and prebiotics can be improved by utilizing the protective and controlled-release properties of hydrogels, which will ultimately lead to more potent microbiome-targeted therapies.

### 6.3. Stability Within Hydrogel Matrices

The efficacy of encapsulation-based delivery systems is significantly influenced by the stability of probiotics and prebiotics within hydrogel matrix [243]. Sensitive biological components are shielded from external stresses including temperature changes, oxygen exposure, and gastrointestinal issues by the protective milieu that hydrogel matrices offer. The shelf life and functional effectiveness of microbiome modulators are greatly increased by this protective action [244].

Probiotics are especially susceptible to environmental deterioration during preparation and storage since they are live bacteria. By forming a physical barrier that restricts exposure to oxygen and moisture, two factors that might lower bacterial viability, hydrogel encapsulation lessens these hazards. Long-term cellular integrity is preserved by the hydrated structure of hydrogels, which also contributes to the maintenance of an environment favorable to bacterial life [245].

The mechanical and chemical characteristics of the matrix also have an impact on the stability of probiotics in hydrogels. The structural integrity of the hydrogel and its capacity to hold onto encapsulated microorganisms are significantly influenced by the crosslinking density, polymer composition, and pore size. Probiotics are guaranteed to remain viable during storage and to be released efficiently once they reach the intended location in the gastrointestinal system when these parameters are optimized [246].

Although not being living things, prebiotics gain from hydrogel encapsulation by being shielded from unchecked fermentation and premature deterioration. The efficiency of prebiotics as substrates for good gut bacteria may be diminished due to their susceptibility to enzymatic degradation and environmental exposure [247].

As a stabilizing medium, hydrogel matrices regulate prebiotic release and guarantee their availability in the gut environment, where they perform their beneficial functions. In hydrogel systems, polysaccharides like pectin and inulin work well to improve stability while preserving prebiotic activity [243].

Other factors to take into account in hydrogel-based encapsulation are temperature and pH stability. Hydrogels intended for gastrointestinal distribution must be able to tolerate the stomach’s acidity and the intestines’ fluctuating pH levels without degrading too quickly. In order to overcome this difficulty, stimuli-responsive hydrogels maintain their stability in the stomach environment while releasing their payload in reaction to enzymatic or intestinal pH triggers. In addition to maintaining stability, this regulated reaction improves therapeutic efficacy and targeted administration [248].

Despite these benefits, it is nevertheless technically difficult to maintain long-term stability within hydrogel matrices. Over time, performance may be impacted by elements such microbial viability loss, structural deterioration, and hydrogel dehydration. The goal of research in this field is to improve storage conditions and material compositions in order to increase scalability and stability. Composite hydrogel and smart material innovations combine mechanical strength and responsive release mechanisms to provide potential solutions [249].

To sum up, hydrogel matrices offer encapsulated probiotics and prebiotics a stable and secure environment, resolving significant drawbacks with direct administration. Hydrogel-based technologies improve the stability and efficacy of treatments that target the microbiome by maintaining biological functions and permitting regulated release. It is anticipated that additional developments in material science will enhance stability even more, opening the door for more reliable and expandable delivery systems [117].

### 6.4. Protection and Controlled Release Considerations in Hydrogel Encapsulation of Probiotics, Prebiotics, and Synbiotics

Overcoming the difficulties presented by the hostile gastrointestinal environment is crucial for the efficient delivery of microbiome modulators like probiotics, prebiotics, and synbiotics. When these compounds reach their intended locations in the intestinal tract, the stomach’s acidic pH, digestive enzymes, and bile salts can drastically lower their viability and usefulness. Because they can protect these bioactives and allow for regulated, targeted release, hydrogels have become promising encapsulating matrices. Therefore, while developing hydrogel-based delivery systems for microbiome modulators, it is crucial to take the significant protection and release factors into account [250].

Hydrogel encapsulation’s main function is to protect probiotics and prebiotics from chemical and physical stresses when they are taken orally. Bile salts and digestive enzymes can further break down probiotics or prematurely break down prebiotics, while the acidic gastric environment (pH ~ 1.5–3.5) might kill or harm these microorganisms.Probiotics are physically isolated by hydrogels’ hydrated barrier, which lowers their vulnerability to low pH and enzymatic attack. Gel beads made of materials like alginate expand in the stomach but do not easily dissolve, forming a protective microenvironment [243,251]. Hydrogels are frequently altered or coated to improve protection; for example, covering alginate beads with chitosan, a polycationic polymer, increases resistance to acidic environments and stops the encapsulated payload from leaking too soon.

Encapsulation also helps prebiotics by preventing early fermentation in the upper gastrointestinal tract, which can lead to adverse effects like gas or bloating. Prebiotics can selectively boost healthy microorganisms in the colon with the help of controlled protection. Combinations of probiotics and prebiotics, known as synbiotics, necessitate a two-pronged defense. The encapsulating matrix must maintain the integrity of the prebiotic substrate while safeguarding live microorganisms. This guarantees that upon release, both parts work in concert [252].

Taking use of the pH variations throughout the digestive tract is a key tactic. In the relatively neutral to slightly alkaline environment of the small intestine or colon, hydrogels can be designed to breakdown or swell while remaining stable in the acidic stomach. For instance, pH-sensitive polymers like carboxymethyl cellulose or pectin enable prompted release in the intestine, guaranteeing that probiotics or prebiotics are supplied exactly where they can colonize or have an impact. Another method for regulated release is enzymatic breakdown. Hydrogels composed of substances broken down by colonic enzymes, such as particular polysaccharides, guarantee payload release in the colon. When prebiotics and synbiotics target the microbiota of the large intestine, this site-specific release is particularly crucial. Additionally, the release profile can be adjusted by changing the bead size, polymer composition, and hydrogel crosslink density. In general, higher crosslink density prolongs release by slowing diffusion. To prevent premature release or inadequate delivery, mechanical strength and permeability must be balanced [253].

Hydrogel-based delivery solutions for microbiome modulators must take protection and regulated release into account. It is feasible to protect probiotics and prebiotics from gastrointestinal stresses and transport them precisely to their target areas through clever hydrogel composition and structural design. The efficacy of probiotic, prebiotic, and synbiotic therapy will be increased by further research into responsive materials and multifunctional hydrogels, opening the door to better gut health interventions.

When creating hydrogel-based probiotic and prebiotic systems, release kinetics is crucial since it dictates when and where the active ingredients are administered in the gastrointestinal tract [240]. For a system to be effective, microbiome modulators must be protected in the acidic gastric environment and allowed to be released under controlled conditions in the intestine or colon, where their activity is needed [254].

In hydrogel matrices, diffusion, swelling, and matrix degradation primarily control release. Crosslinking density and pore size affect diffusion; less dense structures provide rapid diffusion, whereas heavily crosslinked networks offer slower, sustained release. As gastrointestinal fluids are absorbed by hydrogels, they expand and release their encapsulated contents gradually, a process known as swelling-controlled release. The pH of the surrounding environment and the content of the polymers affect the swelling behavior [255].

Additionally, under intestinal circumstances, biodegradable polymers like pectin, chitosan, and alginate can gradually degrade, facilitating targeted release. Furthermore, by staying stable in the stomach and releasing their payload in reaction to microbial enzymes or changes in intestinal pH, pH- or enzyme-responsive hydrogels improve precision. All things considered, maximizing relea*se kinetics necessitates striking a balance between adequate protection and prompt bioavailability. In order to maximize bacterial survival and prebiotic efficacy, carefully planned hydrogel systems can guarantee site-specific, regulated distribution [256].

#### 6.4.1. Comparative Suitability for Hydrogel Incorporation (Natural Antioxidants, Prebiotics, Probiotics, and Combined Systems in Cancer Therapy)

In cancer treatment, hydrogels are promising delivery systems for bioactive substances like probiotics, prebiotics, and natural antioxidants. Through modifying oxidative stress and microbiome interactions, hydrogels may improve treatment results. The nature of the substance, biocompatibility, and controlled release characteristics all affect their applicability [243]. Chitosan, alginate, and hyaluronic acid are examples of natural hydrogels that are beneficial because of their high biocompatibility and capacity to maintain bioactivity under mild manufacturing settings. Sensitive antioxidants and probiotics can be non-covalently encapsulated using these materials, preventing degradation and enabling prolonged release. Particularly in lowering oxidative stress and encouraging immunological modulation, their inherent bioactivity and cell-interactive qualities enable cancer applications [257]. However, the limited mechanical strength and variable disintegration rates of natural hydrogels may limit their long-term usefulness. Long-term functioning may be restricted by the limited mechanical strength and variable degradation rates of natural hydrogels. These constraints can be overcome by improving structural stability and tunability through chemical modification or hybridization with synthetic polymers [258].

Polyethylene glycol (PEG), polyvinyl alcohol (PVA), and poly (2-hydroxyethyl methacrylate) (PHEMA) are examples of synthetic hydrogels that offer better control over mechanical properties and release kinetics. Due to their adjustable network structures, porosity and degradation rates can be precisely adjusted, allowing for consistent and extended therapeutic delivery. For applications needing controlled medication release and structural consistency, synthetic systems are especially well-suited. However, they typically do not have inherent bioactivity and might need to be functionalized to make them more compatible with probiotics and other naturally occurring bioactive substances [149].

Synthetic hydrogels can be designed to react to pathogenic stimuli, such as changes in pH or increased reactive oxygen species (ROS), in cancer therapy. This enhances therapeutic efficacy by enabling site-specific release inside the tumor microenvironment.

The benefits of both natural and synthetic polymers are combined in hybrid hydrogels, which offer controlled release, mechanical strength, and biocompatibility. Given that they preserve microbial viability while guaranteeing the continuous release of bioactive chemicals, these systems are particularly well suited for the co-delivery of probiotics and antioxidants [259]. Hybrid hydrogels provide multimodal treatment approaches in oncology by affecting tumor-associated microbiome, lowering oxidative stress, and modifying immune responses. Multiple therapeutic functions increase their eligibility for combinatorial and individualized treatment techniques. Additionally, hybrid systems facilitate tissue regeneration after tumor excision, meeting both therapeutic and reconstructive goals [260].

Through preserving microbial viability and permitting controlled release in target areas like the gastrointestinal tract or tumor microenvironment, hydrogel encapsulation of prebiotics and probiotics offers important benefits. Prebiotics support immunological control and microbiome modulation by acting as substrates for advantageous bacteria populations. Because hydrogel matrices protect probiotics from hostile environments, their survival and therapeutic potential are enhanced. In cancer treatment, these characteristics are important because the composition of the microbiome has been connected to immune system activation and treatment response. Delivery methods based on hydrogel may improve the stability and effectiveness of therapies that modify the microbiome, leading to better therapeutic results [261].

Antioxidant-prebiotic and probiotic systems are a promising approach to multifunctional cancer treatments. Prebiotics and probiotics support immunological regulation and microbiome homeostasis, while natural antioxidants reduce oxidative stress linked to tumor growth and treatment-induced tissue damage. These drugs can be delivered together in a controlled way thanks to hydrogel platforms, which maximize therapeutic synergy and reduce systemic exposure. In oncology, injectable or localized hydrogels are very useful for site-specific delivery because they improve local bioavailability and minimize off-target effects [262].

In contrast, synthetic hydrogels provide better control over mechanical properties and release kinetics, whereas natural hydrogels are better for applications needing excellent biocompatibility and microbial preservation. Through combining bioactivity and structural stability, hybrid systems offer the most versatility, which makes them appropriate for intricate treatment approaches incorporating antioxidants and drugs that modify the microbiome. These systems enable the dual goals of tissue healing and tumor suppression in cancer therapy [263].

All things considered, hydrogel-based delivery technologies show a great deal of promise for adding probiotics, prebiotics, and natural antioxidants to oncology. By customizing material composition and functionalization techniques, therapeutic effectiveness can be optimized, facilitating the targeted and long-term distribution of bioactive substances. Because of their versatility, hydrogels hold great promise for improving cancer treatment and microbiome-focused therapies.

#### 6.4.2. Critical Comparison of Injectable, Implantable, and Oral Gel-Based Systems in Microbiome-Targeted Cancer Therapy

Gel-based delivery systems are appealing platforms for microbiome-targeted cancer therapy because of their controlled release, biocompatibility, and adjustable mechanical characteristics. However, their efficacy varies significantly according on the administration method [156]. 

Microbial metabolites, immunomodulators, or modified microorganisms can be released locally and sustainably thanks to injectable hydrogels’ less invasive administration and in situ gelation. They offer good spatial control, but unless administered intraluminally, they have little ability to alter the gut microbial environment. Dilution effects and rapid clearance could lessen long-term microbiota remodeling [264].

Implantable gels, also known as pre-formed scaffolds or depots, are especially useful for intratumoral bacterial therapy or localized immune regulation because they provide better local retention and longer release kinetics. Nevertheless, they are less appropriate for systemic microbiome alteration and necessitate invasive insertion [265].

For direct gut microbiota regulation, oral gel technologies (such as pH-responsive microgels) are ideal. Through the gut–tumor axis, they facilitate immunological conditioning and ecological integration by protecting microbial cargo during gastric transit and facilitating colon-specific release. Reproducibility, however, may be impacted by variations in colonization efficiency and gastrointestinal conditions [266].

In general, oral gels are best for modifying the microbiome at the ecosystem level, injectable gels for localized therapy that requires less invasive procedures, and implantable gels for long-term intratumoral use. Therefore, the choice should be in line with the targeted treatment strategy (localized tumor targeting versus ecological reprogramming) [267].

Despite these benefits, there is still insufficient critical discourse regarding the following topics: evaluating which systems are most appropriate for microbiome-targeted cancer therapy; comparing injectable and implantable systems; and clinical translation preparedness. There is currently no systematic assessment of how well they operate in terms of immune modulation capacity, microbiological viability, retention time, geographic precision, and patient compliance [268]. Implantable platforms might provide better control over long-term intratumoral colonization and continuous metabolite administration, even if injectable methods are preferred due to their minimally invasive nature. However, there are still few direct head-to-head preclinical comparisons, which makes it challenging to develop evidence-based system selection guidelines [268].

Furthermore, the therapeutic goal greatly influences the choice of delivery platform for microbiome-targeted cancer therapy. Oral gel methods seem more suitable if the objective is to modify the gut flora ecologically and condition the immune system. In contrast, injectable or implanted methods might be more successful for localized tumor regulation, such as boosting intratumoral bacterial treatment or immune activation. Gel design criteria (mechanical strength, degradation rate, porosity, release kinetics) cannot currently be matched with particular microbiome-mediated therapeutic objectives using a standardized framework [269].

Additionally, clinical translation readiness is still not adequately addressed. While several gel-based systems show encouraging preclinical results, issues with large-scale production, sterilizing without sacrificing microbial viability, long-term biosafety, regulatory approval processes, and reproducibility across patient populations still exist [270]. Furthermore, therapeutic response may be impacted by individual microbiome composition variations, which would complicate the design of clinical trials. Future studies should therefore incorporate translational factors, such as Good Manufacturing Practice (GMP) compliance, regulatory classification (drug-device-biologic combinations), and patient stratification techniques, in addition to optimizing material performance [271].

### 6.5. Preclinical and Clinical Evidence on Hydrogel Encapsulation of Probiotics, Prebiotics, and Synbiotics

An increasing amount of preclinical and clinical research supports promising methods for encapsulating probiotics, prebiotics, and synbiotics in hydrogels. These studies show how encapsulation enhances the therapeutic and functional effects of microbiome modulators by improving their survivability, delivery, and efficacy.

Preclinical research, which is frequently carried out in vitro or in animal models, offers crucial insights into the protective properties of hydrogel encapsulation and its influence on the survivability and functionality of microbiome modulators. Probiotics encapsulated in alginate or chitosan-coated alginate hydrogels, for instance, show noticeably greater survival rates under simulated stomach and intestinal settings than free probiotics, according to a number of in vitro experiments. These hydrogels increase the viability and activity of bacteria by shielding them from bile salts and low pH [272].

Studies on animals support these advantages even more. Compared to non-encapsulated controls, rodent models given hydrogel-encapsulated probiotics exhibit enhanced gut colonization, immune response regulation, and decreased inflammation. Prebiotics encapsulated in hydrogels also exhibit delayed fermentation, enabling targeted colon administration and improving gut microbial community regulation [117].

Preclinical testing of synbiotics co-encapsulated in hydrogels has shown demonstrated synergistic effects on metabolic indicators and the composition of the gut bacteria. These findings emphasize how crucial it is for probiotics and prebiotics to be released and protected together [273].

Although the research is promising, longer-term, more comprehensive clinical trials are required to confirm the benefits of hydrogel encapsulation completely. Transforming preclinical results into clinical practice requires standardization of hydrogel compositions, dosage, and evaluation criteria. The possibility of customized microbiome therapeutics, in which hydrogel-based delivery methods are adapted to specific microbial profiles and medical situations, is also being investigated in future study.

The incorporation of intelligent hydrogels with sensitive release capabilities will improve therapeutic accuracy even more [3,63]. Thus, the potential of hydrogel encapsulation to enhance the distribution and efficacy of probiotics, prebiotics, and synbiotics is supported by preclinical and clinical data. By providing protection against gastrointestinal stresses and facilitating targeted release, this strategy tackles important issues in microbiota modulation.

### 6.6. Synergistic Integration of Natural Antioxidants and Microbiome Modulators in Hydrogel Platforms

Integrating several therapeutic approaches has the ability to overcome the drawbacks of traditional treatments, as demonstrated by recent developments in cancer therapy. Co-delivering natural antioxidants and microbiota modulators in stimuli-responsive hydrogel devices is one intriguing strategy [274]. Microbiome modulators can control systemic and local immune responses, reduce inflammation, and enhance the bioavailability and metabolic activity of co-administered agents [275]. Natural antioxidants can also restore redox balance in tumor microenvironments, reducing oxidative stress-induced DNA damage and therapy resistance [13,276,277].

These bioactive substances can work in concert when included into a single hydrogel platform. A three-dimensional, biocompatible network offered by hydrogels shields delicate molecules from early deterioration and permits regulated, site-specific release in response to environmental cues like pH, temperature, or redox conditions [278]. This makes it possible to modify oxidative stress and microbiota composition at the same time, improving treatment effectiveness and lowering systemic toxicity [279]. Additionally, the co-delivery approach can extend the local presence of bioactive drugs and enhance tumor targeting, overcoming the drawbacks of separate delivery methods.

Overall, the incorporation of microbiome modulators and natural antioxidants into multifunctional hydrogel platforms is a next-generation approach to cancer treatment that provides targeted, localized, and complex interventions. Beyond what can be achieved with hydrogel- or microbiome-targeted techniques alone, this approach not only targets the individual therapeutic potential of each component but also takes advantage of their synergistic interactions to improve treatment outcomes.

### 6.7. Translational, Regulatory, and Manufacturing Challenges

Next-generation hydrogels that incorporate natural antioxidants and microbiota modulators encounter significant translational obstacles, despite encouraging preclinical outcomes. Variability in tumor redox balance affects therapeutic efficiency in addition to interindividual microbiome heterogeneity, which is shaped by food, antibiotic exposure, host genetics, and immunological status [280]. The oxidative profile of the tumor microenvironment determines the biological action of natural antioxidants, whereas patient differences exist in microbial engraftment and metabolite synthesis. Reproducibility, patient stratification, and dose optimization are all made more difficult by this dual variability [281]. Such multifunctional platforms nevertheless have complicated regulatory processes. Hydrogels that contain live biotherapeutic products (LBPs) or metabolites produced from microorganisms and bioactive natural chemicals are frequently categorized as combination products, which may be subject to overlapping drug-biologic or drug-device laws. Regulatory agencies demand thorough genomic and safety profiling of microbial components in addition to rigorous evaluation of polymer composition, crosslinking chemistry, degradation kinetics, and biocompatibility [282].

Virulence variables, genetic stability, antimicrobial resistance genes, biodistribution, shedding risk, and possible horizontal gene transfer are all assessed. The burden of proof for clinical approval is significantly increased when several active components are integrated [283].

Another obstacle is the scalability of manufacturing under GMP standards. It is technically difficult to achieve batch-to-batch reproducibility of hydrogel physicochemical parameters, such as mechanical strength, swelling behavior, and degradation rate, while maintaining microbial viability and antioxidant stability. While live microbial components are sensitive to processing stress and incompatible with traditional terminal sterilizing techniques, many natural antioxidants are vulnerable to oxidation, photodegradation, or fast release [284].

As a result, cold-chain logistics, stability optimization, and aseptic manufacturing processes greatly raise the complexity and expense of production. Overall, to facilitate the practical translation of multifunctional antioxidant–microbiome hydrogel platforms in cancer care, the combined constraints of biological heterogeneity, regulatory complexity, and manufacturing scalability must be methodically addressed.

## 7. Future Perspectives and Conclusions

Cancer is still one of the most serious health issues in the world, and traditional treatments are frequently hindered by serious toxicity and the development of resistance. The gut microbiota and oxidative stress have drawn more attention from scientists in recent years as important modulators of cancer progression and treatment outcomes.

The urgent need for novel treatment approaches is highlighted by the fact that dysbiosis of the gut microbiota and increased oxidative stress levels contribute to both the development of cancer and decreased responsiveness to conventional medicines.

Because of their strong antioxidant, anti-inflammatory, and immunomodulatory qualities, natural antioxidants including lycopene, β-glucans, and EGCG are appealing options for cancer treatment. Probiotics, prebiotics, and synbiotics are examples of microbiome modulators that have demonstrated potential in improving host immune responses and reestablishing microbial equilibrium.

However, these bioactive chemicals’ limited bioavailability, chemical instability, and difficulties in achieving efficient, targeted delivery to tumor locations present major obstacles to their clinical utilization. To get around these restrictions, next-generation hydrogels show promise as adaptable delivery systems. Because of their special qualities, natural antioxidants and microbiota modulators can be encapsulated and released under regulated conditions, preventing premature degradation and allowing for site-specific dosing. This strategy may lessen systemic toxicity while improving the integrated chemicals’ bioavailability and therapeutic efficacy.

In order to produce synergistic effects, the multifunctional hydrogels created and discussed here mix biocompatible polymer networks with carefully chosen bioactives. The antioxidant components reduce inflammation and oxidative stress in the tumor microenvironment, which are known to promote tumor growth and resistance. Concurrently, the microbiome modulators help restore gut microbial equilibrium, which is becoming more widely acknowledged as essential for successful immune responses against cancer.

These hydrogels can sustain the release of antioxidants and microbiome-targeting drugs, maintain their biological activity, and support immunological regulation, according to early in vitro research and animal models. Additionally, the hydrogels have good mechanical qualities, biocompatibility, and biodegradability—all of which are crucial for clinical application.

In conclusion, next-generation hydrogels that incorporate microbiome modulators and natural antioxidants offer a very promising, multifaceted strategy for enhancing cancer treatment. They provide a unique platform that tackles important issues in cancer treatment by bridging the gap between bioactive natural ingredients and cutting-edge biomaterial technology.

**Future perspectives:**

Combining microbiome modulators and natural antioxidants as a comprehensive therapeutic approach: Strong natural antioxidants like lycopene, β-glucans, and EGCG work in concert with microbiome modulators like probiotics, prebiotics, and synbiotics to fight cancer by reducing oxidative stress, controlling inflammation, and reestablishing microbial balance. The main pathogenic pathways causing tumor growth and resistance to traditional treatments are addressed by this holistic approach.

Hydrogels of the future as efficient delivery systems: Next-generation hydrogels provide a flexible and biocompatible matrix that can both protect and facilitate the regulated, site-specific release of medicines that target the microbiota and antioxidants. By improving bioavailability and therapeutic efficacy, this delivery system overcomes drawbacks including low bioavailability and poor stability of natural chemicals, offering a potentially useful tool to better cancer treatment.

Robust preclinical and clinical validation is crucial: Although preliminary in vitro and animal research shows that hydrogels integrated with antioxidants and microbiomes have the ability to alter the tumor microenvironment and systemic immunity, thorough preclinical and clinical studies are necessary. By elucidating mechanisms of action, refining formulations, and establishing safety and efficacy profiles, these studies will open the door for successful translation and incorporation into customized cancer regimens.

## Figures and Tables

**Table 1:** Natural antioxidants with anticancer effects—overview.

Natural Antioxidant	Main Source	Primary Anticancer Mechanism	Specific Characteristics/Effects	References
**Epigallocatechin-3-gallate (EGCG)**	Green tea (Camellia sinensis)	ROS modulation, inhibition of signaling pathways (MAPK, PI3K/Akt/mTOR, NF-κB), apoptosis induction	Flavan-3-ol polyphenol with galloyl group; inhibits growth of cancer cells (colon, pancreatic, lung); modulates tumor microenvironment (inhibits VEGF, PD-L1)	[19,20,21,22,23,24,25,26,27,28,29,30,31,32,33,34]
**Lycopene**	Tomatoes (Solanum lycopersicum)	Potent antioxidant, redox homeostasis regulation, inflammation suppression, apoptosis induction	Lipophilic tetraterpene; inhibits proliferation of prostate, breast, and ovarian cancers; sensitizes HeLa cells to cisplatin	[21,35,36,37]
**β-Glucans**	Fungi (Poria cocos, Saccharomyces), cereals (oats, barley)	Activation of innate and adaptive immunity, tumor microenvironment modulation	Structural polysaccharides recognized by PRRs (Dectin-1, CR3, TLR-2); enhance cytokine release, phagocytosis, cytotoxic T-cell activation	[38,39,40,41,42,43]

**Table 2:** Comparison of advanced delivery systems for natural antioxidants (EGCG, lycopene, and β-glucans) in oncology applications.

Parameter	Hydrogels	Nanoparticles	Liposomes	Polymeric Carriers
Structural organization	3D crosslinked hydrophilic polymer networks	Solid colloidal systems (10–200 nm), polymeric or inorganic	Phospholipid bilayer vesicles	Biodegradable synthetic/natural polymers (e.g., PLGA, PEG)
Primary administration strategy	Local or site-specific	Systemic (passive/active targeting)	Systemic	Systemic
Protection against pH degradation (EGCG)	High [20,69]	High [20,69]	Moderate–High [20,69]	High [20,69]
Improvement of membrane permeability	Limited (local diffusion)	High (endocytosis-mediated uptake) [20,69]	Moderate [20,69]	Moderate–High [20,69]
Ability to bypass P-gp efflux	Indirect	Yes [20,48,68,69]	Partial [20,69]	Yes (formulation-dependent) [20,69]
Suitability for lipophilic compounds (lycopene)	Limited [70,71]	Very Good [70,71]	Excellent [70,71]	Very Good [70,71]
Elimination of dietary-fat dependence (lycopene)	Yes [70,73,74]	Yes [70,73,74]	Yes [70,73,74]	Yes [70,73,74]
Controlled/sustained release	Excellent	Good	Moderate	Excellent
Tumor microenvironment targeting	Local implantation possible	EPR effect; ligand functionalization [67,72]	Surface-modifiable	Ligand-functionalizable
Co-delivery capability	Excellent [67]	Good [67]	Moderate	Good [67]
Systemic circulation stability	Low (mainly local use)	High [67,72]	Moderate	High
Clinical development status	Emerging	Preclinical–clinical [67,72]	Clinically established platform	Clinically established polymers
Main limitations	Limited systemic distribution	Potential nanotoxicity	Drug leakage; stability issues	Complex synthesis; cost

**Table 3:** Role of gut microbiome, probiotics, prebiotics, and synbiotics in cancer development and treatment.

Topic	Description	References
**Gut Microbiome and Cancer**	Influences cancer development and treatment outcomes via inflammation, immune modulation, metabolic activity, and genotoxic effects.	[83,84,85,86,87]
**Gut Microbiota Composition**	The human gastrointestinal tract hosts diverse microbes (~10^10^ to 10^12^ per gram in colon) crucial for health maintenance.	[84,85]
**Probiotics**	Live microorganisms that confer health benefits when administered in adequate amounts; proven safe and effective in multiple conditions including cancer prevention.	[90,92,95,96]
**Probiotic Mechanisms**	Suppress harmful microbes, modify gut microbiota composition, stimulate immune responses, and produce metabolites (bacteriocins, amines, H_2_O_2_) that regulate apoptosis, proliferation, inflammation, and differentiation.	[11,88,89]
**Prebiotics**	Non-digestible, selectively fermented short-chain carbohydrates (e.g., fructooligosaccharides, galactooligosaccharides) promoting growth/activity of beneficial bacteria and improving gut physiology.	[90,91,97,98]
**Dietary Sources of Prebiotics**	Naturally present in asparagus, garlic, chicory, onion, wheat, banana, barley, tomato, rye, soy, milk, peas, beans, seaweeds, microalgae, etc.	[97,98]
**Synbiotics**	Combination of probiotics and prebiotics designed to improve probiotic survival and implantation, stimulate beneficial bacteria, and modulate gut metabolism and integrity.	[92,93,94]

**Table 4:** Natural antioxidants incorporated into hydrogel systems for enhanced stability and therapeutic delivery.

Antioxidant/Component	Incorporation Method	Hydrogel Properties and Benefits	Specific Examples and Effects	References
Catechins, Flavonoids, Carotenoids, Polysaccharides	Physical entrapment or chemical conjugation	Physical entrapment preserves bioactivity via non-covalent interactions; chemical conjugation improves stability and retention but requires careful design	Enables control over loading efficiency, stability, release kinetics, and therapeutic effect	[192,193,194]
Base Polymers (N-carboxyethyl chitosan vs. Hyaluronic acid)	Polymer selection impacts gel strength and release	N-carboxyethyl chitosan hydrogels show stronger polymer interactions, enhancing gel strength, prolonging antioxidant retention, and slowing release	Example: Resveratrol release can be tailored by adjusting hydrogel structure	[192,193,194]
Stimuli-responsive Hydrogels	Environment-triggered release (pH, temperature, ROS)	pH-responsive hydrogels target acidic tumor microenvironments; temperature-responsive hydrogels (e.g., Pluronic F127 + chitosan) allow localized in situ gelation	ROS-sensitive hydrogels exploit oxidative stress for controlled release; polyphenols act as physical crosslinkers and metal–phenolic coordinators provide self-healing properties	[195,196,197,198,199,200,201,202,203]
Chemical Crosslinking	Covalent bonding via enzyme crosslinking, free-radical polymerization, click chemistry	Ensures permanent antioxidant integration with enhanced structural stability		[204,205]
EGCG	Incorporated in mucoadhesive NanoCubogels and co-encapsulation with probiotics	Improves stability, bioavailability, prolonged local release, enhanced permeation	Alginate-whey protein isolate hydrogels protect both EGCG and probiotics during storage and GI transit	[206,207]
Lycopene	Encapsulation in alginate hydrogel beads	Overcomes hydrophobicity and instability, improves storage stability and controlled release	Enhances bioaccessibility compared to non-encapsulated forms	[207,208]
β-Glucans	Used as hydrogel matrix or crosslinked with peptides	Enhances gel strength, water retention, thermal stability; shows multifunctional wound healing, antioxidative and anti-inflammatory properties	Oat β-glucan in konjac glucomannan hydrogels; oxidized β-glucan crosslinked with antimicrobial peptides	[209,210]

## Data Availability

No new data were created or analyzed in this study.

## Abbreviations

The following abbreviations are used in this manuscript:

AAc	Acrylic acid
ABTS	2,2′-azinobis (3-ethylbenzothiazoline-6-sulfonic acid)
Bax/Bcl-2	Bcl-2-associated X protein/B-cell lymphoma 2
BRMs	Biological response modifiers
CA125	Cancer antigen 125
Caspase-3	Cysteine-aspartic acid protease 3
Caspase-9	Cysteine-aspartic acid protease 9
CD4^+^	Cluster of differentiation 4 (helper T cells)
CD8^+^	Cluster of differentiation 8 (cytotoxic T cells)
CDK	Cyclin-dependent kinase
CR3	Complement receptor 3
CR3-DCC	Complement-dependent cellular cytotoxicity
CRC	Colorectal cancer
Cx43	Connexin 43
DCs	Dendritic cells
Dectin-1	Dendritic cell-associated C-type lectin-1
DIABLO	Direct IAP-binding protein with low pI
DNA	Deoxyribonucleic acid
DNMT1	DNA methyltransferase 1
DPPH	2,2-diphenyl-1-picrylhydrazyl
EC	Epicatechin
ECM	Extracellular matrix
ECS	Endothelial cells
EGC	Epigallocatechin
EGCG	Epigallocatechin-3-gallate
EMT	Epithelial–mesenchymal transition
ERK1/2	Extracellular signal-regulated kinases 1 and 2
FAO	Food and Agriculture Organization
FMT	Fecal microbiota transplantation
FRAP	Ferric reducing antioxidant power
GelMA	Gelatin methacryloyl
GelMA-CPBA	Gelatin methacryloyl functionalized with 3-(carboxyphenyl)boronic acid
GJC	Gap junctional intercellular communication
GO	Graphene oxide
HA	Hyaluronic acid
HA-SH	Thiolated hyaluronic acid
HIF-1α	Hypoxia-inducible factor-1α
HO-1	Heme oxygenase-1
HPLC	High-performance liquid chromatography
iC3b	Inactive complement component 3b
IGF-1	Insulin-like growth factor 1
IL-6	Interleukin-6
IL-12	Interleukin-12
IPNs	Interpenetrating polymer networks
ISAPP	International Scientific Association of Probiotics and Prebiotics
ISX	Intestine-specific homeobox
IκBα	Inhibitor of kappa B alpha
JNK	c-Jun N-terminal kinase
LNPs	Lipid nanoparticles
M1	Macrophage phenotype (antitumoral)
MAPK	Mitogen-activated protein kinase
MDA	Malondialdehyde
MMP-2/MMP-9	Matrix metalloproteinases 2 and 9
mTOR	Mammalian target of rapamycin
NF-κB	Nuclear factor-kappa B
NK	Natural killer cells
NO	Nitric oxide
NOX4	NADPH oxidase 4
NP	Nanoparticle
Nrf2	Nuclear factor erythroid 2–related factor 2
ORAC	Oxygen radical absorbance capacity
p21Cip1/Waf1	Cyclin-dependent kinase inhibitor 1A
p27KIP1	Cyclin-dependent kinase inhibitor 1B
PAA	Poly (acrylic acid)
PAAm	Poly (acrylamide)
PBS	Phosphate-buffered saline
PDE	Partial differential equation
PD-L1	Programmed death-ligand 1
PEG	Poly (ethylene glycol)
PEO	Poly (ethylene oxide)
P-gp	P-glycoprotein
PheLigNPs	Phenolated lignin nanoparticles
PHEMA	Poly (2-hydroxyethyl methacrylate)
PI3K	Phosphatidylinositol 3-kinase
PRRs	Pattern recognition receptors
PVA	Poly (vinyl alcohol)
ROS	Reactive oxygen species
SCXN	Xylan-based capecitabine complex
SIRT1	Sirtuin 1
Smac	Second mitochondria-derived activator of caspases
SNPs	Single nucleotide polymorphisms
SR-B1	Scavenger receptor class B type 1
Syk	Spleen tyrosine kinase
TA	Tannic acid
TAFs	Tumor-associated fibroblasts
TLA	Three-letter acronym
TLR-2	Toll-like receptor 2
TME	Tumor microenvironment
TNF-α	Tumor necrosis factor-alpha
Tregs	Regulatory T cells
UV	Ultraviolet
VEGF	Vascular endothelial growth factor
VI	1-Vinylimidazole
WHO	World Health Organization
